# Umbilical venous catheter and peripherally inserted central catheter malposition and tip migration in neonates: A mixed methods cost analysis

**DOI:** 10.1016/j.ijnsa.2025.100450

**Published:** 2025-11-10

**Authors:** Arun M Jones, Suzanna Mongan, Amanda Ullman, Deanne August, Elizabeth Sharpe, Angela A Alderman, Darcy Doellman, Caitlin Anders, Kacey Wiseman, Cheryl Gillette, Hansoo Kim, Joshua Byrnes

**Affiliations:** a Health Economics Group, Griffith University, Gold Coast, Australia; b School of Nursing, Midwifery and Social Work, University of Queensland, Queensland, Australia; c Royal Brisbane & Women’s Hospital, Herston, Brisbane, Queensland, Australia; d College of Nursing, The Ohio State University, Columbus, OH, USA; e Neonatal Intensive Care Nursery, Carilion Children’s Hospital, VA, USA; f B Braun Medical Inc. Bethlehem, PA, USA; g Vascular Access Team, Nationwide Children’s Hospital, Columbus, OH, USA; h Vascular Access Service, Boston Children’s Hospital, Boston, MA, USA; i Pediatric Vascular Access Service, University of Rochester Medical Center, Golisano Children’s Hospital, Rochester, NY, USA; j Children’s Health Queensland Hospital and Health Service, Brisbane, Australia

**Keywords:** Costs and cost analysis, Health care economics and organizations, Infant, Newborn / hospitalization, Health care costs, Hospital, Models, Economic, Vascular access devices / adverse effects

## Abstract

**Background:**

Hospitalized neonates require reliable vascular access for life-saving care. The costs associated with their clinical management, and which aspects of care these costs are attributable to, is not well-known.

**Objective:**

To estimate the economic burden of vascular access care in neonates in the United States and to break down the attribution of costs therein by establishing an economic model of standard care.

**Design and methods:**

A four step, mixed-methods study was used to determine and analyse an appropriate economic model for neonatal umbilical venous catheter and peripherally inserted central catheter insertion from the payer’s perspective in the US. An initial model was developed based on a purposive literature search. Secondly, initial face validity of the model was assessed with input from North American clinical experts identified to have appropriate expertise (*n* = 13 for the care of peripherally inserted central catheters and *n* = 12 for the care of umbilical venous catheters).Thirdly, a face-to-face meeting with the same clinical experts was undertaken to ensure the model structure and inputs accurately reflected clinical practice. Lastly, the finalised model was analysed.

**Results:**

Feedback from the survey and focus group on model structure, resource usage and costings were incorporated to create decision-tree models for both umbilical venous catheter and peripherally inserted central catheter care. High variability between the opinions of clinicians was noted, which was incorporated into the sensitivity analyses. The umbilical venous catheter base-case expected cost was $390.24 per patient, with an average of 0.04 complications expected per-patient. The peripherally inserted central catheter model base-case expected cost was $1517.83 per patient, with an average of 0.1 complications per-patient. In the umbilical venous catheter model $82.73 of cost was attributable to malposition and $46.36 to migration. In the peripherally inserted central catheter model, $75.58 was attributable to malposition and $755.14 to migration. Deterministic sensitivity analysis indicated that the strongest driver of costs was catheter dwell time (umbilical venous catheter lower: $245.55, umbilical venous catheter upper: $578.77, peripherally inserted central catheter lower: $1263.40, peripherally inserted central catheter upper: $1771.74), followed by probability of migration (umbilical venous catheter lower: $343.91, umbilical venous catheter upper: $439.14, peripherally inserted central catheter lower: $1329.04, peripherally inserted central catheter upper: $1733.58) in both models.

**Conclusions:**

The migration and malposition of peripherally inserted central catheters and umbilical venous catheters has significant costs and consequences. These should be targeted for evidence-based and innovative solutions to improve neonatal vascular access care.

**What is already known**

• Venous catheters are essential for neonates requiring vascular access.
• Typical methods for determining catheter positioning often result in complications.
• The costs of managing catheters in neonatal care is poorly quantified.

Alt-text: Unlabelled box

**What this paper adds**

• In neonates, we find the average per-patient cost of a peripherally inserted central catheter and umbilical venous catheter to be $1,518 and $390 respectively.
• In neonates, migration and malposition make up 55 % of total peripherally inserted central catheter costs, and 33 % of umbilical venous catheter costs.
• Migration and malposition are identified as areas of high cost in the usage of peripherally inserted central and umbilical venous catheters in neonates, interventions which target migration and malposition would be likely to significantly reduce complications and costs.

Alt-text: Unlabelled box

## 1 Background

Vascular access is frequently required in hospitalized neonates for various short- and long-term needs. Establishing reliable vascular access is crucial as a life-saving procedure for neonates requiring immediate care. However, obtaining vascular access in neonates can be challenging due to small and mobile veins, making visualization and palpation of veins difficult (Detaille et al., 2010; Scott-Warren and Morley, 2015). The choice of vascular access device used depends on the patient’s clinical condition, skin condition, treatment duration, and properties of the infusate (Nakamura et al., 2020; Scott-Warren and Morley, 2015). Umbilical venous catheters are more generally used in cases for immediate, short term central access (CDC, 2022), whilst peripherally inserted central catheters are used when longer-term central access is required (Sharpe et al., 2024). More recent studies have highlighted the role of clinician preference and practice inertia plays in choice of venous access devices, and have called for and propose more standardised guidelines to guide such decisions (Barone et al., 2023; van Rens et al., 2024).

Umbilical venous catheters (hereafter referred to as an umbilical catheters) and neonatal peripherally inserted central catheters (hereafter referred to as a central catheters) are two of the most frequently inserted devices in neonatal units, with potential complications occurring at insertion or during dwell (Dongara et al., 2017). Umbilical catheters are the predominant vascular access devices for significantly unwell newborns globally (Saugstad et al., 2021). The umbilical catheter is inserted into the umbilical vein of the neonatal umbilical cord and the catheter tip should reside in the inferior vena cava at or just superior to the diaphragm and below the right atrium (Nickel et al., 2024). The traditional methods (Sharma et al., 2019) for determining the proper length of insertion, such as Dunn’s (Dunn, 1966) or Shukla and Ferrara’s (Shukla and Ferrara, 1986) often result in incorrect positioning (Kieran et al., 2016), and securing the catheters is challenging due to factors such as desiccating tissue, angle of insertion, and limited securement area (Nakamura et al., 2020; Oestreich, 2010; Stuttaford et al., 2022; Perl et al., 2024).

Neonatal central catheters can be inserted using upper extremity or scalp veins with the desired catheter tip location being the lower third of the superior vena cava. Neonatal central catheters are also referred to as epicutaneocava catheters in some regions outside of the US (van Rens et al., 2024), however we opted to use the central catheter nomenclature to maintain consistency with clinicians’ naming conventions in the study setting. Neonatal central catheters may be inserted through lower extremity veins such as the great saphenous for eventual catheter tip location in the inferior vena cava above the level of the diaphragm (Nickel et al., 2024; Sharpe et al., 2024). An umbilical catheter or central catheter is considered malpositioned if its tip does not terminate in the appropriate location at the time of insertion (Sharpe et al., 2024).

Typically, confirmation of catheter tip location for umbilical and central catheters are achieved through chest X-rays, although ultrasonography can also be used (Scott-Warren and Morley, 2015). X-rays have drawbacks, such as radiation exposure, possible misinterpretation of tip location, and variable image quality (Connolly et al., 2006; Gnannt et al., 2016). Ultrasonography provides the advantage of reducing exposure to harmful radiation whilst being more accurate than X-rays (Cao et al., 2023; Oleti et al., 2019). However, its use is restricted by its expense and practicality; one literature review concluded that ultrasound should be a complement to, instead of a replacement for x-rays (Nguyen, 2016).

Healthcare professionals caring for neonates face various challenges when performing umbilical and central catheter insertions, including limited time due to competing interests, limited resources, and varying skill levels (D'Andrea et al., 2023). It has been estimated that 40 % to 45 % of umbilical catheterss are inaccurately placed on the first attempt, leading to multiple re-attempts or even failure to establish access (Hoellering et al., 2018; Hall et al., 2025; Mutlu et al., 2017).

Migration occurs when the catheter tip location changes from a satisfactory documented post-insertion location to a different location after initial placement (Sharpe et al., 2024). Catheter tip migration is frequently reported in the literature (Dubbink-Verheij et al., 2019; Hoellering et al., 2018; Nadroo et al., 2002), and while migration may in some cases be easily managed by adjusting the length, it may quickly escalate and cause severe complications such as hepatic extravasation, cardiac tamponade, bloodstream infections, and delay in delivering appropriate treatment (Ullman et al., 2015; Chen et al., 2020; Kurtom et al., 2016).

Traditional length estimation using surface landmarks for central catheters results in approximately a one in three misplacement rate among newborns (Zhou et al., 2017). When multiple insertion attempts are made, it can lead to multiple vein punctures, which can damage delicate vessels, deplete available veins and increase the risk of complications such as infection and thrombosis (Gnannt et al., 2018). One US study estimated a central catheter migration incidence of 28 % (Acun et al., 2021), the resulting complications of which are a significant concern in paediatric populations (Nadroo et al., 2002; Badheka et al., 2019; Ares and Hunter, 2017).

Amidst rising healthcare costs and increased emphasis on value-based care that aims to improve patient outcomes whilst restraining costs, it is imperative that the potentially avoidable harm and costs from malposition and migration are understood. Quantifying these costs paramount for informing future resource allocation decisions and facilitating broader discussion over the problem (Greenberg, 2014). This study aims to determine the financial burden associated with the insertion of both umbilical and central catheters in neonates by synthesising evidence from existing literature with the knowledge of North American clinical experts. We account for the potential costs incurred due to malposition and migration associated complications, which will enable us to breakdown the attribution of burden.

## 2 Methods

### 2.1 Design and setting

A four-step mixed-method study analysing the costs and outcomes of umbilical and central catheters in neonates in a US NICU setting from the perspective of the payer was undertaken.

• Step 1: Initial model development.
• Step 2: Initial face-validity of model structure and model input parameter estimates via electronic survey (Supplement 1).
• Step 3: Confirmatory face-validity and finalisation of model parameter estimates (via face-to-face focus group and follow-up survey).
• Step 4: Analysis.

*Step 1: Initial model development*

Two decision-analytic models were built in Treeage Pro Healthcare 2024 (TreeAge Software, 2024), a modelling software which can be used to calculate the expected costs and outcomes associated with healthcare interventions. Potential pathways associated with the intervention are delineated and attributed associated costs and outcomes. The weighted average of the costs and outcomes of each potential pathway are deemed the expected costs and outcomes. The payer’s perspective was chosen as it aligns with that of stakeholders who make resource allocation decisions. A time-horizon equal to the catheter’s dwell time was chosen since this captures all the costs and outcomes associated with vascular access care.

An initial decision tree structure was developed based on input from authors with clinical experience. A targeted, purposive search of existing literature on PubMed and Google Scholar was used to populate the model with input parameters. Sources were chosen based on their relevance to model structure and evidence quality. Where no published evidence was found for input parameters, proxies or assumptions were used in place. Cost parameters for initial model were sourced from the Australian Pharmaceutical Benefits Scheme (Australian Government: Department of Health, 2025b) or Medicare Benefits Scheme (Australian Government: Department of Health, 2025a), since the US lacks any such centralised database of health resource costs. These were then updated or affirmed by the clinician responses to surveys. Any foreign currency costs were first inflated and converted (adjusting for purchasing power parity) from their reported source currencies to 2024 US dollars ($) using a cost converting tool (Shemilt et al., 2010). Where incidence rates were reported in the literature, they were converted to a transition probability (a probability determining the likelihood of following one branch of the tree compared to another) according to $p=1-e^{-rt}$, where r is the incidence rate and t is the model time-horizon (Briggs et al., 2006). Otherwise, proportions of patients in which the outcome occurs was used directly to be the parameter value. The output of this step was a plausible initial model to serve as a starting point for validation by clinical expert opinion.

*Step 2: Initial face-validity of model structure and model input parameter estimates via electronic survey*

Participants based in the US with suitable clinical expertise, defined as at least five years of experience in vascular access with a significant component of experience in a neonatal setting, were identified within professional networks, face-to-face meetings, email and online social media campaigns. A member of the research team informed participants of the research project and provide them with time to consider the information and ask questions. A copy of the information sheet and consent form were given to the participant, and the original securely stored by research staff for 7 years from project conclusion.

The participants were sent an electronic survey (Supplement 1) including the initial version of the decision tree and a brief explanation of the model; and asked to provide feedback with respect to the model structure, including identification of specific complications associated with malposition and migration, resources associated with insertion, positioning, location confirmation and treatment of possible complications.

*Step 3: Confirmatory face-validity and finalisation of model parameter estimates*

A face-to-face meeting was held where a summary of the initial feedback from the electronic survey was presented. A discussion facilitated by XXXX was then held to confirm and adjust the structure of the model to reflect clinical practice. The focus group was recorded and transcribed. Following this, a subsequent version of each of the peripheral and umbilical catheter models along with the specific data input parameters was circulated to all participants with an opportunity for further comment and to provide feedback.

*Step 4: Analysis*

Upper and lower estimates of each input parameter were confirmed from clinical experts. No discount rate was employed as the time-horizon used (umbilical catheter: 4 days, central catheter: 14 days) is short enough such that it would not make any meaningful difference.

The primary outcome was expected cost per patient in 2024 US dollars, which was estimated along with the expected count of all malposition, reposition and migration associated complications per patient, giving the average number of complications experienced per patient (hereafter referred to as expected complications). Treeage Pro Healthcare 2024 (TreeAge Software, 2024) was used to calculate the expected cost and expected complications for the base case, which uses estimates of transition probabilities and costs that reflect current clinical practice. Three separate scenario analyses checked the model results where the following inputs were changed from the base case:

1) Probability of malposition is equal to zero
2) Probability of migration is equal to zero
3) Probability of migration and malposition is equal to zero

To estimate the expected cost attributable to malposition, the expected cost from the zero-malposition scenario is subtracted from the expected cost from the base case. This is also done to find the cost attributable to migration, and the cost of malposition and migration combined, in both models.

Deterministic sensitivity analysis was undertaken for both models. The model was re-run with the upper and lower bound values of each model input parameter to see how these affect the model outputs. The results of this analysis were graphically represented using a tornado diagram. No health economic analysis plan was developed prior to undertaking the analysis.

### 2.2 Ethical considerations

Ethical approval for the focus group and survey was received from the Griffith University Human Research Ethics Committee (reference number: 2023/617).

## 3 Results

*Step 1: Initial model development*

The initial model structure was informed entirely by the authorship team’s knowledge of clinical care pathways. The purposive review identified articles reporting the incidence of our initially identified complications relating to umbilical and central catheter use. Sources which were incorporated into the model are summarised in supplementary Table S1.

*Step 2: Initial face-validity of model structure and model input parameter estimates via electronic survey*

A total of 22 clinical experts were invited with 13 participating in the initial survey. One participant provided feedback only with respect to the central catheter model as they self-identified as having no experience with umbilical catheters. Participants were granted anonymity as a condition of taking part to encourage open discussion. Participants included nine vascular access specialists, one NICU team leader, one chief nursing officer, one clinical professor, and one vascular access team leader. Eleven participants were vascular access board certified and four Certified Neonatal Intensive Care Nurses. Experience of respondents ranged between 5–44 years with a mean of 24.6 years. Geographic representation of respondents came from five different states (Arizona, California, New York, Massachusetts and Ohio).

Based on the initial feedback, potential changes to the model structure were identified as well as changes to the range of values for the input parameters, based on the participants’ clinical experience and subjective judgement.

*Step 3: Confirmatory face-validity and finalisation of model parameter estimates*

All the 13 clinical experts who participated in the initial survey took part in the subsequent focus group. During the facilitated discussion, the identified changes to the decision tree were considered and a revised decision tree was developed. With respect to the input parameters, discussion was focused on the extent to which the upper and lower estimates captured the likely heterogeneity across sites. Model input values were subsequently updated according to group consensus with respect to the base value as well as the upper and lower bounds.

Feedback was received from four participants to the post focus group survey. Based on this, no changes to the model structure were made but adjustments of model input parameters were undertaken.

In the umbilical catheter model, the following variables had their upper and lower bounds expanded: probability of catheter reposition (15 %−35 % to 0 %−50 %), probability of switching to a central catheter (85 %−95 % to 80 %−100 %), and umbilical catheter dwell time (2–6 days to 1–7 days).

In the central catheter model, migration was reduced from 47 % to 28 % and probability of neurological complications was reduced from 5 % to 3.5 %. The following variables had their upper and lower bounds expanded: probability of catheter reposition (56 %−93 % to 50 %−100 %), probability of catheter reinsertion (37 %−62.5 % to 30 %−70 %), probability of complications from extravasation (3.2 %−4 % to 1 %−6.2 %).

*Step 4: Analysis*

### 3.1 Final model structure

**Fig. 1:**
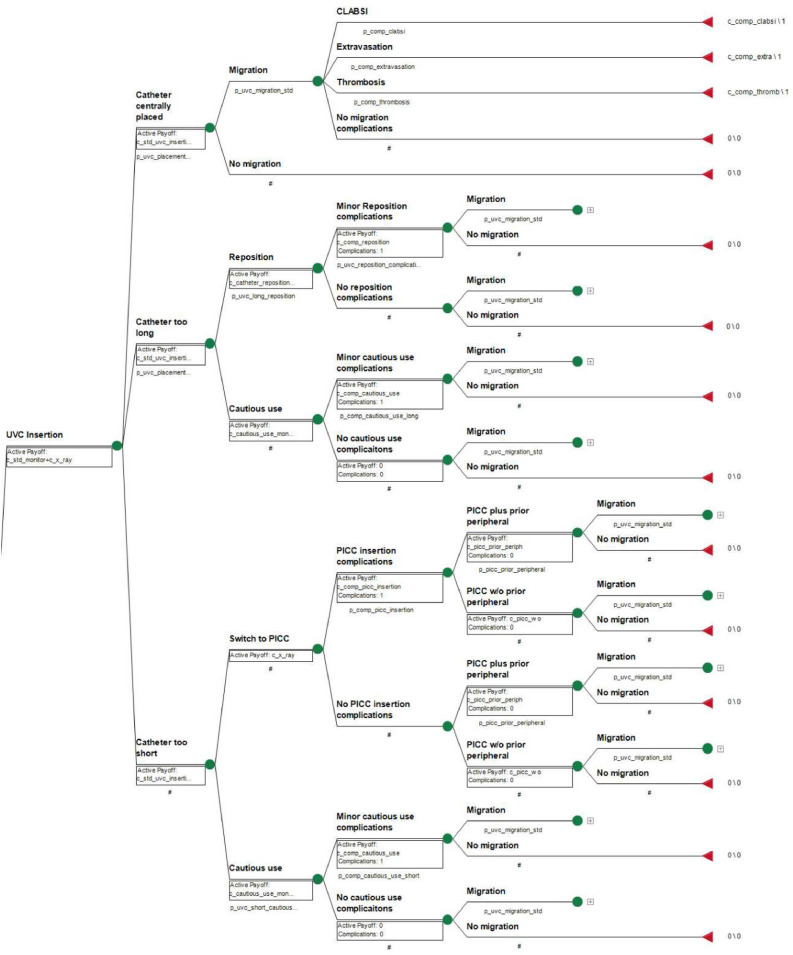
Umbilical venous catheter model diagram.

**Fig. 2:**
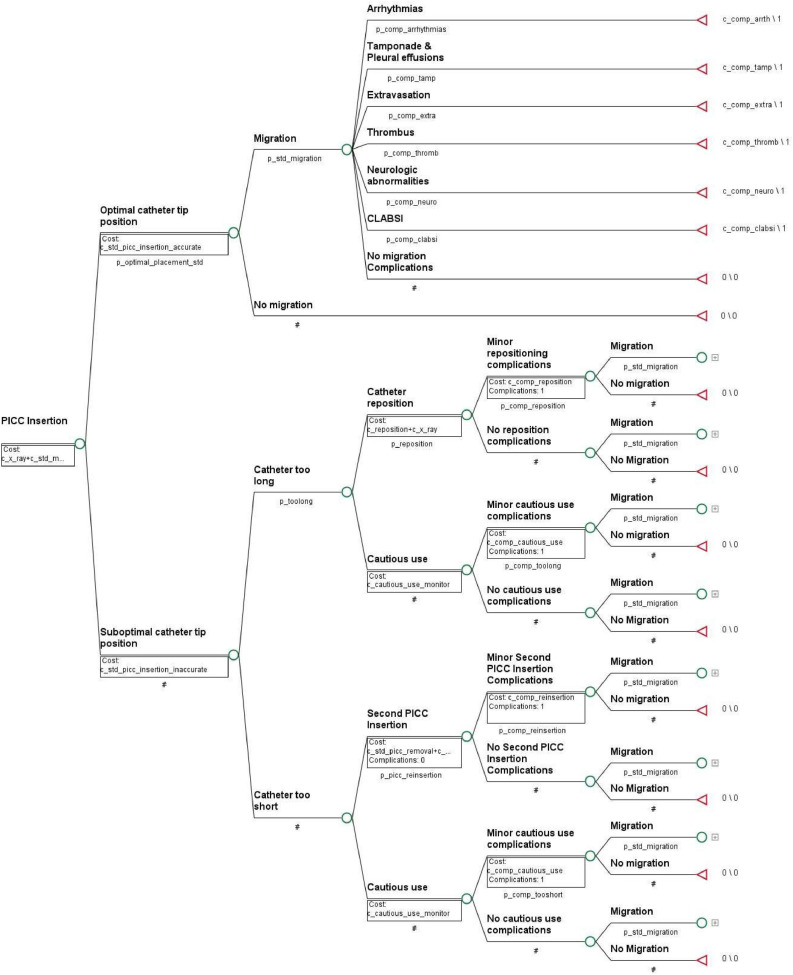
Central catheter model diagram.

The final umbilical and central catheter model structures are shown in Fig. 1, Fig. 2 respectively. Note that in the model figure, nodes following catheter-tip migration are only shown in the first instance they are encountered, and then collapsed thereafter for brevity. Both models start at initial catheter insertion, where the earliest nodes (to the left) determine the initial catheter tip position as either optimally placed or sub-optimally placed on initial insertion, following a post-insertion x-ray for tip location confirmation.

Fig. 1 legend: Each circle represents a chance node with a probability of multiple outcomes; the triangle represents the terminal node where the model ends; the [+] represents a collapsed branch equivalent in structure to one presented elsewhere in the model.

Fig. 2 Legend: Each circle represents a chance node with a probability of multiple outcomes; the triangle represents the terminal node where the model ends; the [+] represents a collapsed branch equivalent in structure to one presented elsewhere in the model.

A catheter tip placed optimally then faces the risk of migrating over the remainder of the model time-horizon. If at any point in the model a catheter tip does migrate, then the patient faces the risk of encountering catheter migration-related complications, or no complications occurring, all of which subsequently terminate the model.

Should the catheter tip be considered too long, the clinician may determine if the catheter tip is repositioned or used cautiously with extra monitoring. Unspecified minor complications may arise directly because of repositioning or cautious use, following which the catheter tip runs the risk of migrating.

If a catheter tip was determined to be too short on initial insertion, the clinician is to decide whether the short catheter tip is either used cautiously with extra monitoring, or if the original catheter is removed and a new central catheter inserted (replacing the umbilical catheter in the umbilical catheter model). If a subsequent central catheter was to be inserted, the following nodes depend on the model in question. In the umbilical catheter model, the patient then runs the risk of encountering minor central catheter insertion complications, and the costs of inserting the central catheter depends on whether the catheter was inserted with or without a prior temporary peripheral device, the new catheter then runs the risk of migrating. In the central catheter model, the patient faces the risk of minor complications arising from the second central catheter insertion, and then the catheter runs the risk of migrating. If the short initially placed catheter tip were to be used cautiously instead, the model follows the same subsequent pathways as a long catheter tip used cautiously as described for a catheter tip found to be too long.

### 3.2 Input parameters

The value for each input parameter in the final model, along with the lower and upper estimates used in the deterministic sensitivity analysis, are presented in Table 1. Where the source is labelled “Survey” the parameter values were informed by our face-to-face focus group and/or follow-up survey. Parameters with a published source listed were affirmed by the clinical experts in the face-to-face focus group and/or follow-up survey.

**Table 1:** Umbilical and central catheter model input parameters.

Model	Parameter	Value	Lower bound	Upper bound	Source
*Transition Probabilities*					
Umbilical	Probability of repositioning a catheter found to be too long	25 %	0 %	50 %	Survey
Umbilical	Probability of switching to central catheter after catheter is found to be too short	90 %	80 %	100 %	Survey
Umbilical	Probability of switching to central catheter with prior peripheral	50 %	20 %	80 %	Survey
Central	Probability of catheter reinsertion after catheter is found to be too short	50 %	30 %	70 %	Survey
Central	Probability of repositioning a catheter found to be too long	75 %	50 %	100 %	Survey
Central	All-cause probability of catheter line associated bloodstream infection	2.3 %	1.9 %	2.7 %	Milstone et al.(Milstone et al., 2013)
Umbilical	All-cause probability of catheter line associated bloodstream infection	1.4 %	1.0 %	2.1 %	Gibson et al.(Gibson et al., 2021)
Both	Proportion of all-cause catheter line associated bloodstream infections attributable to migration	9.67 %	4.67 %	14.67 %	Salzman et al.(Salzman and Rubin, 1997) & survey
Umbilical	Probability of umbilical catheter tip being placed too deep/far on initial insertion	17 %	15 %	19 %	Franta et al.(Franta et al., 2017)
Umbilical	Probability of umbilical catheter tip being positioned optimally on initial insertion	58 %	52 %	63 %	Wu et al.(Wu et al., 2020)
Central	Probability of a sub optimally placed catheter being too long (as opposed to too short)	87 %	65 %	100 %	Jain et al.(Jain et al., 2013)
Central	Probability of arrythmia	1.2 %	0.9 %	1.5 %	Dhillon et al.(Dhillon et al., 2020)
Central catheter	Probability of cardiac tamponade	1.8 %	1.6 %	2.0 %	Pezzati et al.(Pezzati et al., 2004)
Umbilical	Probability of complications from central catheter insertion when switching to central catheter	5.0 %	3.8 %	6.3 %	Survey
Central	Probability of complications from central catheter reinsertion	2.0 %	1.5 %	2.5 %	Survey
Both	Probability of complications from catheter reposition	0.1 %	0.1 %	0.1 %	Survey
Umbilical	Probability of complications from cautious use after catheter misplaced (too long)	10 %	8 %	13 %	Survey
Central	Probability of complications from cautious use after catheter misplaced (too long)	87 %	65 %	100 %	Survey
Both	Probability of complications from cautious use after catheter misplaced (too short)	30 %	23 %	38 %	Survey
Central	Probability of extravasation	3.6 %	1.0 %	6.2 %	McIntyre et al.(McIntyre et al., 2023)
Umbilical	Probability of extravasation	0.1 %	0.0 %	0.1 %	Gibson et al.(Gibson et al., 2021)
Umbilical	Probability of migration	14 %	0 %	29 %	Gibson et al.(Gibson et al., 2021)
Central	Probability of migration	28 %	21 %	36 %	Acun et al.(Acun et al., 2021)

Model	Parameter	Value	Lower	Upper	Source
Central	Probability of neurological complications	3.5 %	1.0 %	5.0 %	Survey
Central	Probability of optimal catheter position on initial insertion	79 %	70 %	88 %	Ling et al.(Ling et al., 2019)
Umbilical	Probability of thrombosis	2.1 %	0.0 %	4.2 %	Gibson et al.(Gibson et al., 2021)
Central	Probability of thrombosis	9.2 %	6.9 %	11.5 %	Park et al.(Park et al., 2014)
*Resource Cost*					
Umbilical	Umbilical catheter Dwell time (days)	4	1	7	Sanderson et al.(Sanderson et al., 2017)
Central	Central catheter Dwell time (days)	14	7	21	Milstone et al.(Milstone et al., 2013)
Umbilical	Cost of central catheter insertion with prior peripheral	$178.78	$134.08	$223.48	Australian MBS item 13,319(Online, 2024a)
Umbilical	Cost of central catheter insertion without prior peripheral	$198.78	$149.08	$248.48	Survey
Central	Cost of central catheter reposition	$178.78	$134.08	$223.48	Survey
Umbilical	Cost of umbilical catheter reposition	$70.66	$53.00	$88.33	Survey
Central	Cost of accurate central catheter insertion	$178.78	$160.90	$196.65	Australian MBS item 13,319(Online, 2024a)
Umbilical	Cost of accurate umbilical catheter insertion	$116.59	$87.44	$145.74	Survey
Both	Cost of an x-ray	$27.36	$24.62	$30.09	Australian MBS item 58,500(Online, 2024b)
Both	Cost of complications from cautious use of misplaced catheter	$70.66	$53.00	$88.33	Survey
Central	Cost of complications from second central catheter insertion	$70.66	$53.00	$88.33	Survey
Central	Cost of inaccurate central catheter insertion				Survey
Umbilical	Cost of inaccurate umbilical catheter insertion	$138.08	$103.56	$172.60	Survey
Central	Cost of removing a central catheter	$70.66	$63.59	$77.73	Survey
Both	Cost of treating catheter line associated bloodstream infection	$76,464.78	$38,169.25	$114,760.31	Goudie et al.(Goudie et al., 2014)
Central	Cost of treating arrythmias	$6255.16	$4691.37	$7818.95	Wodchis et al.(Wodchis et al., 2012)
Central	Cost of treating cardiac tamponade	$76,276.25	$68,627.47	$106,460.19	Iribarne et al.(Iribarne et al., 2012)
Umbilical	Cost of treating complications from central catheter insertion when switching to central catheter	$70.66	$53.00	$88.33	Survey
Both	Cost of treating complications from catheter reposition	$70.66	$53.00	$88.33	Survey
Both	Cost of treating extravasation	$2651.37	$1988.53	$3314.21	Hanrahan(Hanrahan, 2013)
Central	Cost of treating neurological complications	$351.66	$263.75	$439.58	Survey
Both	Cost of treating thrombosis	$10,548.73	$9493.86	$11,603.61	Haddad et al.(Haddad et al., 2014)
Central	Additional daily cost of cautious use & monitoring after catheter inserted in suboptimal location	$29.31	$21.98	$36.64	Survey
Umbilical	Additional daily cost of cautious use & monitoring after catheter inserted in suboptimal location	$29.31	$21.98	$36.63	Survey

Model	Parameter	Value	Lower bound	Upper bound	Source
Both	Daily monitoring costs	$29.31	$21.98	$36.63	Survey
Both	Hourly wage of consulting neonatologist	$57.51	$60.39	$54.63	Queensland Health(Health, 2024c)
Both	Hourly wage of health practitioner (level 1)	$23.27	$24.43	$22.11	Queensland Health(Health, 2024b)
Both	Hourly wage of health practitioner (level 5)	$50.46	$52.98	$47.94	Queensland Health(Health, 2024b)
Both	Hourly wage of nurse practitioner	$53.23	$55.89	$50.57	Queensland Health(Health, 2024a)
Both	Hourly wage of registered nurse	$30.59	$32.12	$29.06	Queensland Health(Health, 2024a)

### 3.3 Expected cost and complications associated malposition and migration for umbilical and central catheters

In the umbilical catheter model, we find the base-case expected cost to be $390.24 USD ($) per patient (divided by four days gives $97.57 per patient per day), with expected complications at 0.04 events (Table 2). There is a large reduction in expected cost and complications when we switch to the zero-malposition scenario ($307.54 and 0.003 respectively). In the no migration scenario, we estimate expected cost at $343.91, with expected complications at 0.03. As expected, the no migration and malposition scenario results in the lowest expected cost of $261.18 with zero malposition and migration associated complications.

**Table 2:** Expected malposition and migration associated complication costs (USD 2024) per patient for umbilical catheters.

	Umbilical Catheter	
Scenario	Complications (all)	Cost
Base Case	0.03506	$390.24
Zero malposition	0.00322	$307.54
Zero migration	0.03184	$343.91
Zero migration and malposition	0	$261.18
*Attributable cost: malposition*		$82.73
*Attributable cost: migration*		$46.36
*Attributable cost: malposition and migration*		$129.09

In the central catheter model, the base case expected cost is $1517.83 per patient (divided by fourteen days gives $108.42 per patient per day), with 0.1 expected complications (Table 3). In the no malposition scenario, expected costs are $1442.25 and expected complications 0.054, whilst in the no migration scenario, expected costs are $762.69 and expected complications 0.044. In the no malposition and migration scenario, central catheter costs are $687.11, with zero complications. In contrast to the umbilical catheter model, the expected cost here is lower in the no migration scenario when compared to the no malposition scenario. This is due to the skew in the proportion of complications attributable to migration versus malposition between both models. In the central catheter model, 55 % of complications are migration related, whilst only 9 % are in the umbilical catheter model. This is a result of the probability of migration-related complications being modelled as an increasing function of catheter dwell time. Initial insertion malposition-related complications, conversely, are static with respect to dwell time. Total attributable cost of malposition and migration is estimated to be $830.73 in the central catheter model and $129.09 in the umbilical catheter model. The total cost attributable to malposition is $75.58 in the central catheter model and $82.73 in the umbilical catheter model, whilst the total cost attributable to migration is $755.14 in the central catheter model and $129.09 in the umbilical catheter model.

**Table 3:** Expected malposition and migration associated complication costs (USD 2024) per patient for central catheters.

	Central Catheter	
Scenario	Complications (all)	Cost
Base Case	0.0995	$1517.83
Zero malposition	0.0548	$1442.25
Zero migration	0.0447	$762.69
Zero migration and malposition	0	$687.11
*Attributable cost: malposition*		$75.58
*Attributable cost: migration*		$755.14
*Attributable cost: malposition and migration*		$830.73

### 3.4 Sensitivity analysis

**Fig. 3:**
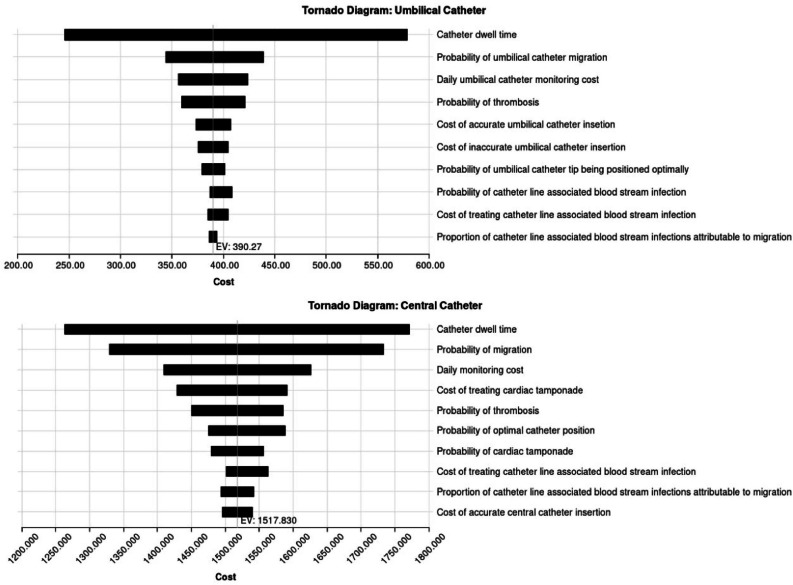
Deterministic sensitivity analysis results.

Both models are similar in that they share the same variables within their top three individual drivers of expected cost (Fig. 3). Namely, catheter dwell time, probability of catheter migration, and monitoring costs. These do not come as a surprise since dwell time strictly increases both monitoring costs and the probability of more costly migration-related complications, whilst the increased probability of catheter migration increases the occurrence of migration-related complications. The uncertainty in the relationship between malposition and migration is also reflected with the wide range between the upper and lower estimates tested. More detailed sensitivity analysis results are displayed in supplementary Table S2.

Fig. 3 legend: EV – Expected value.

## 4 Discussion

This study has established a model of the cost associated with catheter malposition and migration among neonates requiring umbilical and central catheters in a US hospital setting. Combined costs attributable to malposition and migration of $830.73 and $129.09 per patient for central and umbilical catheter insertion respectively demonstrate the consequence of imperfect care and highlight the potential for cost reduction from strategies which target malposition and migration. For central catheters, the expected cost attributable to migration was greater than that of malposition whereas in the umbilical catheter model initial malposition was more costly (central catheter: $755.14 vs. $75.58; umbilical catheter: $53.89 vs $82.73). This establishes the timepoints (insertion or management) where change and innovation are most needed. Cost analyses such as these can inform cost effectiveness analyses and drive adoption in cost-effective technologies, improving resource allocation within neonatal intensive care units (NICU).

Until now, there have been limited studies examining costs of vascular access care (Comber et al., 2024), especially in neonates. A cost analysis on paediatric patients estimated central catheter costs at $374 per day (Dong et al., 2018), whilst one specifically on neonates estimated daily central catheter cost at $81 per day (Chenoweth et al., 2018). Our base case estimate of $108 per day lies on the lower-end of this range. A retrospective cost-effectiveness analysis estimated daily umbilical catheter cost at $57 (Guzmán-de la Garza et al., 2020), in contrast with this study’s estimate of $98 per day. Other adjacent analyses estimate related factors such as burden specific to central catheter failure but excluding cost of routine care (Ullman et al., 2022) or broader statewide costs (Tuffaha et al., 2019). Adjacent economic evaluations tend to exclude neonatal patients (Calvert et al., 2003; Periard et al., 2008). Comparable analyses to this study often suffer to varying degrees from a lack of generalisability due to being single-centre studies. The present study aims to alleviate this by incorporating wide upper and lower bounds for parameter inputs, which cover the opinions of clinicians from many different centres across the US. However, these are still limited to the US perspective. As such, the transferability of these results in international settings is dependent on how well our final input parameters translate internationally, although our ranges of results identified in the sensitivity analyses could be considered transferrable to some extent, especially across high-income countries. One strength of this methodology is that such a model can easily be re-run with new input parameters to obtain more location-specific results, provided that the model structure itself still reflects clinical practice.

A clear limitation of this model is the use of foreign costing data in cases where US values were not available. While affirmation of these converted costs by the surveyed clinicians provides some degree of validation, nevertheless this is an imperfect solution due to the relatively small sample size of clinicians available to us.

Another limitation of this study is attributed to the nature of the evidence found in existing literature concerning the parameters utilized in the model, necessitating reliance on the insights of clinical experts. Moreover, variations in the management, treatment and outcomes of neonatal umbilical and central catheters are notable across jurisdictions and NICUs. Despite efforts to mitigate this in the model by incorporating appropriate upper and lower bounds in the sensitivity analyses, it remains uncertain whether this comprehensively encompasses the actual impact of parameter uncertainty on our estimates and the extent to which this reflects specific local settings.

One avenue for future research could be the validation of these results through comparison with real-world data, such as hospital billing data. This could be done from a US perspective or otherwise, validating domestically and internationally. If deviations are found in input parameter values, the model can be re-run with new parameters to match the locality.

The high financial costs of infection complications due to central vascular access devices remain a significant concern in healthcare (Ullman et al., 2022; Wilson et al., 2014; Karagiannidou et al., 2019; Goudie et al., 2014). However, the financial implications of other migration and malposition-related complications do contribute additional burden and should also be a focus of improvement and innovation (Goudie et al., 2014). Deterministic sensitivity analysis demonstrated that the catheter dwell time, probability of catheter migration, and monitoring costs are the largest drivers of expected cost in both catheter types. Interventions targeting these areas could therefore be good candidates for reducing financial burden. The significant drop in expected costs and complications in the absence of migration and malposition scenarios underscores the potential for not only improved patient outcomes but substantive and healthcare cost efficiencies. A promising innovation that could change the consequence of complications at insertion and dwell could be the utilization of intracavitary electrocardiography tip location devices. These devices demonstrate the potential to decrease both insertion and post-insertion complications while also offering healthcare providers alerts for tip migration (Ullman et al., 2022; Kleidon et al., 2021; Yuan et al., 2017; Xiao et al., 2020).

## 5 Conclusion

The migration and malposition of umbilical and central catheters in neonates can have significant consequences for overall costs, neonatal and family outcomes. Our cost-analysis demonstrates that minimizing migration and malposition issues can lead to cost reduction and improved patient outcomes. However, there remains significant uncertainty around the probability of migration, its associated complications, and the model’s reliance on the opinion of clinical experts both structurally and with respect to its parameters. It is crucial to prioritize the prevention of migration and malposition as a key objective in the routine care of neonates in the NICU, with a need for evidence-based solutions to address these.

## Funding

This study was funded in part by an investigator-initiated grant from Navi Medical Technologies Pty Ltd.

## Data statement

The study did not use external or proprietary data sources. All data underlying the results are fully presented in the manuscript tables and figures. Readers can reconstruct the dataset and replicate the analyses from the material contained in the article.

## CRediT authorship contribution statement

**Arun M Jones:** Writing – review & editing, Writing – original draft, Visualization, Validation, Software, Methodology, Investigation, Formal analysis, Data curation, Conceptualization. **Suzanna Mongan:** Writing – review & editing, Writing – original draft, Investigation. **Amanda Ullman:** Writing – review & editing, Writing – original draft, Validation, Supervision, Resources, Project administration, Methodology, Investigation, Funding acquisition, Data curation, Conceptualization. **Deanne August:** Writing – review & editing, Writing – original draft, Visualization, Validation, Supervision, Resources, Project administration, Methodology, Investigation, Data curation, Conceptualization. **Elizabeth Sharpe:** Writing – review & editing, Validation, Methodology, Investigation. **Angela A Alderman:** Writing – review & editing, Validation, Methodology, Investigation. **Darcy Doellman:** Writing – review & editing, Validation, Methodology, Investigation. **Caitlin Anders:** Writing – review & editing, Validation, Methodology, Investigation. **Kacey Wiseman:** Writing – review & editing, Validation, Methodology, Investigation. **Cheryl Gillette:** Writing – review & editing, Validation, Methodology, Investigation. **Hansoo Kim:** Writing – review & editing, Writing – original draft, Supervision, Software, Resources, Project administration, Methodology, Investigation, Formal analysis. **Joshua Byrnes:** Writing – review & editing, Writing – original draft, Visualization, Validation, Supervision, Software, Resources, Project administration, Methodology, Investigation, Funding acquisition, Formal analysis, Data curation, Conceptualization.

## Declaration of competing interest

The authors declare the following financial interests/personal relationships which may be considered as potential competing interests: Joshua Byrnes reports financial support was provided by Navi Medical Technologies. Joshua Byrnes reports a relationship with Bard Peripheral Vascular Inc that includes: funding grants. Joshua Byrnes reports a relationship with 3 M Company that includes: funding grants.

Darcy Doellman reports a relationship with B.Braun Medical that includes: employment.

Elizabeth Sharpe reports a relationship with Argon Medical Devices Inc that includes: consulting or advisory.

Amanda Ulman reports investigator-initiated research grants paid to her employer from BD, 3 M, Medline and Biolife, unrelated to this study.

Hansoo Kim has received research funding or consulting fees from Abbott, BD, CSL, Edwards LifeSciences, Moderna, Novartis, Sanofi and UCB.

If there are other authors, they declare that they have no known competing financial interests or personal relationships that could have appeared to influence the work reported in this paper.

## Appendix Supplementary materials

Image, application 1

Image, application 2

Image, application 3

## References

[bib0003] Acun C., Baker A., Brown L.S., Iglesia K.A., Sisman J. (2021). Peripherally inserted central cathether migration in neonates: incidence, timing and risk factors. J. Neonatal. Perinat. Med..

[bib0004] Ares G., Hunter C.J. (2017). Central venous access in children: indications, devices, and risks. Curr. Opin. Pediatr..

[bib0005] Australian Government: Department of Health, Disability and Ageing (2025). https://www.mbsonline.gov.au/.

[bib0006] Australian Government: Department of Health, Disability and Ageing (2025). https://www.pbs.gov.au/pbs/home.

[bib0007] Badheka A., Bloxham J., Schmitz A., Freyenberger B., Wang T., Rampa S., Turi J., Allareddy V., Auslender M., Allareddy V. (2019). Outcomes associated with peripherally inserted central catheters in hospitalised children: a retrospective 7-year single-centre experience. BMJ Open..

[bib0008] Barone G., D’andrea V., Ancora G., Cresi F., Maggio L., Capasso A., Mastroianni R., Pozzi N., Rodriguez-Perez C., Romitti M.G., Tota F., Spagnuolo F., Raimondi F., Pittiruti M. (2023). The neonatal DAV-expert algorithm: a GAVeCeLT/GAVePed consensus for the choice of the most appropriate venous access in newborns. Eur. J. Pediatr..

[bib0009] Briggs A., Sculpher M., Claxton K. (2006).

[bib0010] Calvert N., Hind D., Mcwilliams R.G., Thomas S.M., Beverley C., Davidson A. (2003). The effectiveness and cost-effectiveness of ultrasound locating devices for central venous access: a systematic review and economic evaluation. Health Technol. Assess.

[bib0011] Cao J., Zhang Y., Yin Y., Liu Y. (2023). Accuracy of chest radiography compared to ultrasound for positioning the umbilical venous catheter in neonates: a meta-analysis and systematic review. J. Vasc. Access.

[bib0012] CDC (2022). Prevention and Control of Infections in Neonatal Intensive Care Unit Patients: central Line-associated Blood Stream Infections. Infect. Control.

[bib0013] Chen H.-J., Chao H.-C., Chiang M.-C., Chu S.-M. (2020). Hepatic extravasation complicated by umbilical venous catheterization in neonates: a 5-year, single-center experience. Pediatr. Neonatol..

[bib0014] Chenoweth K.B., Guo J.W., Chan B. (2018). The extended dwell peripheral intravenous catheter is an alternative method of NICU intravenous access. Adv. Neonatal. Care.

[bib0015] Comber E.R., Keogh S., Nguyen L.N., Byrnes J., Ullman A.J. (2024). Implementation frameworks, strategies and outcomes in optimizing central venous access device practice in paediatrics: a scoping review. J. Adv. Nurs..

[bib0016] Connolly B., Amaral J., Walsh S., Temple M., Chait P., Stephens D. (2006). Influence of arm movement on central tip location of peripherally inserted central catheters (PICCs). Pediatr. Radiol..

[bib0017] D'andrea V., Prontera G., Pinna G., Cota F., Fattore S., Costa S., Migliorato M., Barone G., Pittiruti M., Vento G. (2023). Securement of umbilical venous catheter using cyanoacrylate glue: a randomized controlled trial. J. Pediatr..

[bib0018] Detaille T., Pirotte T., Veyckemans F. (2010). Vascular access in the neonate. Best Pract. Res. Clin. Anaesthesiol..

[bib0019] Dhillon S.S., Connolly B., Shearkhani O., Brown M., Hamilton R. (2020). Arrhythmias in children with peripherally inserted central catheters (PICCs). Pediatr. Cardiol..

[bib0020] Dong Z., Connolly B.L., Ungar W.J., Coyte P.C. (2018). Cost analysis of peripherally inserted central catheter in pediatric patients. Int. J. Technol. Assess. Health Care.

[bib0021] Dongara A.R., Patel D.V., Nimbalkar S.M., Potana N., Nimbalkar A.S. (2017). Umbilical venous catheter versus peripherally inserted central catheter in neonates: a randomized controlled trial. J. Trop. Pediatr..

[bib0022] Dubbink-Verheij G.H., Visser R., Tan R., Roest A.A.W., Lopriore E., Te Pas A.B. (2019). Inadvertent migration of umbilical venous catheters often leads to malposition. Neonatology.

[bib0023] Dunn P.M. (1966). Localization of the umbilical catheter by post-mortem measurement. Arch. Dis. Child.

[bib0024] Franta J., Harabor A., Soraisham A.S. (2017). Ultrasound assessment of umbilical venous catheter migration in preterm infants: a prospective study. Arch. Dis. Child. - Fetal Neonatal Ed..

[bib0025] Gibson K., Sharp R., Ullman A., Morris S., Kleidon T., Esterman A. (2021). Adverse events associated with umbilical catheters: a systematic review and meta-analysis. J. Perinatol..

[bib0026] Gnannt R., Connolly B.L., Parra D.A., Amaral J., Moineddin R., Thakor A.S. (2016). Variables decreasing tip movement of peripherally inserted central catheters in pediatric patients. Pediatr. Radiol..

[bib0027] Gnannt R., Waespe N., Temple M., Amirabadi A., Liu K., Brandao L.R., Connolly B.L. (2018). Increased risk of symptomatic upper-extremity venous thrombosis with multiple peripherally inserted central catheter insertions in pediatric patients. Pediatr. Radiol..

[bib0028] Goudie A., Dynan L., Brady P.W., Rettiganti M. (2014). Attributable cost and length of stay for central line-associated bloodstream infections. Pediatrics.

[bib0029] Greenberg P.E. (2014). Cost of illness: an ongoing battle worth fighting. Pharmacoeconomics.

[bib0030] Guzmán-De La Garza F.J., Laredo-Flores A.D., Cárdenas-Del Castillo B., Cordero-Franco H.F., Salinas-Martínez A.M., Fernández-Garza N.E., Ochoa-Correa E. (2020). Ultrasound-guided umbilical venous catheterisation: a cost-effectiveness analysis. An. Pediatr. (Engl. Ed.).

[bib0031] Haddad H., Lee K.-S., Higgins A., Mcmillan D., Price V., El-Naggar W. (2014). Routine surveillance ultrasound for the management of central venous catheters in neonates. J. Pediatr..

[bib0032] Hall S., Larsen E., Cobbald L., Marsh N., Mclaughlin L., Takashima M., Ware R.S., Ulman A., August D. (2025). Recurrent peripheral intravenous catheterization in neonates: a case series. Nurs. Crit. Care.

[bib0033] Hanrahan K. (2013). Hyaluronidase for treatment of intravenous extravasations: implementation of an evidence-based guideline in a pediatric population. J. Spec. Pediatr. Nurs..

[bib0034] Health, Q. (2024).

[bib0035] Health, Q. (2024).

[bib0036] Health, Q. (2024).

[bib0037] Hoellering A., Tshamala D., Davies M.W. (2018). Study of movement of umbilical venous catheters over time. J. Paediatr. Child Health.

[bib0038] Iribarne A., Burgener J.D., Hong K., Raman J., Akhter S., Easterwood R., Jeevanandam V., Russo M.J. (2012). Quantifying the incremental cost of complications associated with mitral valve surgery in the United States. J. Thorac. Cardiovasc. Surg.

[bib0039] Jain A., Deshpande P., Shah P. (2013). Peripherally inserted central catheter tip position and risk of associated complications in neonates. J. Perinatol..

[bib0040] Karagiannidou S., Zaoutis T., Maniadakis N., Papaevangelou V., Kourlaba G. (2019). Attributable length of stay and cost for pediatric and neonatal central line-associated bloodstream infections in Greece. J. Infect. Public Health.

[bib0041] Kieran E.A., Laffan E.E., Donnell C.P.F. (2016). Estimating umbilical catheter insertion depth in newborns using weight or body measurement: a randomised trial. Arch. Dis. Child. - Fetal Neonatal Ed..

[bib0042] Kleidon T.M., Horowitz J., Rickard C.M., Ullman A.J., Marsh N., Schults J., Ratz D., Chopra V. (2021). Peripherally inserted central catheter thrombosis after placement via electrocardiography vs traditional methods. Am. J. Med..

[bib0043] Kurtom W., Quast D., Worley L., Oelberg D.G. (2016). Incorrect umbilical vein catheterization is associated with severe periventricular hemorrhages and mortality in extremely premature newborns. J. Neonatal. Perinat. Med..

[bib0044] Ling Q., Chen H., Tang M., Qu Y., Tang B. (2019). Accuracy and safety study of intracavitary electrocardiographic guidance for peripherally inserted central catheter placement in neonates. J. Perinat. Neonatal. Nurs..

[bib0045] Mcintyre C., August D., Cobbald L., Lack G., Takashima M., Foxcroft K., Marsh N., Smith P., New K., Koorts P. (2023). Neonatal vascular access practice and complications: an observational study of 1375 catheter days. J. Perinat. Neonatal. Nurs..

[bib0046] Milstone A.M., Reich N.G., Advani S., Yuan G., Bryant K., Coffin S.E., Huskins W.C., Livingston R., Saiman L., Smith P.B., Song X. (2013). Catheter dwell time and CLABSIs in neonates with PICCs: a multicenter cohort study. Pediatrics.

[bib0047] Mutlu, M., Parıltan, B.K., Aslan, Y., Eyüpoğlu, İ., Kader, Ş. & Aktürk, F.A.J.T.A.O.P.T.P.A. 2017. Comparison of methods and formulas used in umbilical venous catheter placement. 52**,** 35. 10.5152/TurkPediatriArs.2017.4912 PMC5396820 28439199

[bib0048] Nadroo A.M., Glass R.B., Lin J., Green R.S., Holzman I.R. (2002). Changes in upper extremity position cause migration of peripherally inserted central catheters in neonates. Pediatrics.

[bib0049] Nakamura H., Nakamura R., Paran T.S. (2020). Vascular access in infants and children. Pediatr. Surg..

[bib0050] Nguyen J. (2016). Ultrasonography for central catheter placement in the neonatal intensive care unit—a review of utility and practicality. Am. J. Perinatol..

[bib0051] Nickel B., Gorski L., Kleidon T., Kyes A., Devries M., Keogh S., Meyer B., Sarver M.J., Crickman R., Ong J., Clare S., Hagle M.E. (2024). Infusion therapy standards of practice, 9th edition. J. Infus. Nurs..

[bib0052] Oestreich A.E. (2010). Umbilical vein catheterization—appropriate and inappropriate placement. Pediatr. Radiol..

[bib0053] Oleti T., Jeeva Sankar M., Thukral A., Sreenivas V., Gupta A.K., Agarwal R., Deorari A.K., Paul V.K. (2019). Does ultrasound guidance for peripherally inserted central catheter (PICC) insertion reduce the incidence of tip malposition? – a randomized trial. J. Perinatol..

[bib0054] Online, M. (2024). https://www9.health.gov.au/mbs/fullDisplay.cfm?type=item&q=13319&qt=item.

[bib0055] Online, M. (2024). https://www9.health.gov.au/mbs/fullDisplay.cfm?type=item&q=58500&qt=item.

[bib0056] Park C.K., Paes B.A., Nagel K., Chan A.K., Murthy P. (2014). Neonatal central venous catheter thrombosis: diagnosis, management and outcome. Blood Coagul. Fibrinolysis.

[bib0057] Periard D., Monney P., Waeber G., Zurkinden C., Mazzolai L., Hayoz D., Doenz F., Zanetti G., Wasserfallen J.B., Denys A. (2008). Randomized controlled trial of peripherally inserted central catheters vs. peripheral catheters for middle duration in‐hospital intravenous therapy. J. Thromb. Haemost..

[bib0058] Perl J.R., Crabtree-Beach T., Olyaei A., Hedges M., Jordan B.K., Scottoline B. (2024). Reducing umbilical catheter migration rates by using a novel securement device. J. Perinatol..

[bib0059] Pezzati M., Filippi L., Chiti G., Dani C., Rossi S., Bertini G., Rubaltelli F.F. (2004). Central venous catheters and cardiac tamponade in preterm infants. Intensive Care Med..

[bib0060] Salzman M.B., Rubin L.G. (1997). Relevance of the catheter hub as a portal for microorganisms causing catheter-related bloodstream infections. Nutrition.

[bib0061] Sanderson E., Yeo K., Wang A., Callander I., Bajuk B., Bolisetty S., Lui K., Bowen J., Sedgley S., Carlisle H. (2017). Dwell time and risk of central-line-associated bloodstream infection in neonates. J. Hosp. Infect..

[bib0062] Saugstad O.D., Robertson N.J., Vento M. (2021). A critical review of the 2020 international liaison committee on resuscitation treatment recommendations for resuscitating the newly born infant. Acta Paediatr..

[bib0063] Scott-Warren V.L., Morley R.B. (2015). Paediatric vascular access. BJA Educ..

[bib0064] Sharma D., Farahbakhsh N., Tabatabaii S.A. (2019). Role of ultrasound for central catheter tip localization in neonates: a review of the current evidence. J. Matern.-Fetal Neonatal Med..

[bib0065] Sharpe E.L., Curry S., Wyckoff M.M. (2024). NANN neonatal peripherally inserted central catheters: guideline for practice, 4th ed. Adv. Neonatal Care.

[bib0066] Shemilt I., James T., Marcello M. (2010). A web-based tool for adjusting costs to a specific target currency and price year. Evid. Policy.

[bib0067] Shukla H., Ferrara A. (1986). Rapid estimation of insertional length of umbilical catheters in newborns. Am. J. Dis. Child..

[bib0068] Stuttaford L., Webb J., Smith S.L., Powell C., Watkins W.J., Chakraborty M. (2022). Estimating insertion length of umbilical arterial and venous catheters in newborn infants: time for change. J. Matern. Fetal. Neonatal. Med..

[bib0069] TREEAGE SOFTWARE 2024. TreeAge Pro Healthcare 2024. Williamstown, MA.

[bib0070] Tuffaha H.W., Marsh N., Byrnes J., Gavin N., Webster J., Cooke M., Rickard C.M. (2019). Cost of vascular access devices in public hospitals in Queensland. Aust. Health Rev..

[bib0071] Ullman A.J., Gibson V., Takashima M.D., Kleidon T.M., Schults J., Saiyed M., Cattanach P., Paterson R., Cooke M., Rickard C.M., Byrnes J., Chopra V. (2022). Pediatric central venous access devices: practice, performance, and costs. Pediatr. Res.

[bib0072] Ullman, A.J., Marsh, N., Mihala, G., Cooke, M. & Rickard, C.M.J.P. 2015. Complications of central venous access devices: a systematic review. 136**,** e1331–e1344. 10.1542/peds.2015-1507 26459655

[bib0073] Van Rens M.R., Van Der Lee R., Spencer T.R., Van Boxtel T., Barone G., Crocoli A., Pinelli F., Pittiruti M. (2024). The NAVIGATE project: a GloVANet-WoCoVA position statement on the nomenclature for vascular access devices. J. Vasc. Access..

[bib0074] Wilson M.Z., Rafferty C., Deeter D., Comito M.A., Hollenbeak C.S. (2014). Attributable costs of central line-associated bloodstream infections in a pediatric hematology/oncology population. Am. J. Infect. Control.

[bib0075] Wodchis W.P., Bhatia R.S., Leblanc K., Meshkat N., Morra D. (2012). A review of the cost of atrial fibrillation. Value Health.

[bib0076] Wu L., Peng M., Cao T., Yang Y., Wang Q., Luo K., Chen P. (2020). Application of a modified electrocardiogram-guided technique for umbilical venous catheterisation in neonates: a retrospective trial. J. Paediatr. Child Health.

[bib0077] Xiao A.-Q., Sun J., Zhu L.-H., Liao Z.-Y., Shen P., Zhao L.-L., Latour J.M. (2020). Effectiveness of intracavitary electrocardiogram-guided peripherally inserted central catheter tip placement in premature infants: a multicentre pre-post intervention study. Eur. J. Pediatr.

[bib0078] Yuan L., Li R., Meng A., Feng Y., Wu X., Yang Y., Chen P., Qiu Z., Qi J., Chen C., Wei J., Qin M., Kong W., Chen X., Xu W. (2017). Superior success rate of intracavitary electrocardiogram guidance for peripherally inserted central catheter placement in patients with cancer: a randomized open-label controlled multicenter study. PLoS. One.

[bib0079] Zhou L., Xu H., Liang J., Xu M., Yu J. (2017). Effectiveness of intracavitary electrocardiogram guidance in peripherally inserted central catheter tip placement in neonates. J. Perinat. Neonatal. Nurs..

