# Supplementary material for: Umbilical venous catheter and peripherally inserted central catheter malposition and tip migration in neonates: A mixed methods cost analysis
Source: Int J Nurs Stud Adv. 2025 Nov 10;9:100450. doi: 10.1016/j.ijnsa.2025.100450 (PMC12664355; doi:10.1016/j.ijnsa.2025.100450)
Supplement: Supplementary file 1 [file mmc1.pdf]

Griffith UNIVERSITY
Centre for Applied Health Economics

Supplement 1

Pre-Focus Group Activity: Model confirmation

PICC and UVC insertion in Neonates

Introduction

Thank you for agreeing to participate in this study.

Pre-focus Group Activity Aim:

- As you are a clinical expert, we would like to seek your feedback on our economic models to evaluate potential benefits from new technologies that aim to improve umbilical venous catheter (UVC) and peripherally inserted central catheter (PICC) insertion for neonates and reduce the risk of migration during dwell.
- The overall aim of this pre-focus group exercise is to validate our model(s) to ensure they represent standard practice in a USA hospital setting.

Economic Model:

- Our methodology utilises a decision tree to perform analyses. Each tree model represents a simplified patient journey starting from the insertion of a central catheter (PICC or UVC) in a neonate.
- The patient can move across the branches of the decision tree where at each node a patient could move down different clinical pathways until reaching the end of a pathway .
- The models were created to include what could happen to reach successful insertion, and capture complications associated with catheter tip migration during dwell.

What is a decision tree and why?

- A decision tree is commonly used for economic assessment within the healthcare sector. It is a predictive model that uses a flowchart-like structure to make decisions based on input data.

- This technique is applied in situations where the outcomes of an event are uncertain, yet it is feasible to assign probabilities to various outcomes.

- We understand in a clinical setting, patients and cases are not so straightforward and predictable. However, to conduct the analysis, we needed to create linear pathways to simplify the process.

If you would like, we are more than happy to walk through the model with you one-on-one and answer any questions.

Email: Josh Byrnes (j.byrnes@griffith.edu.au) to find a time that suits.

Pre-focus group activity

- Prior to the focus group on the 14th October, we would like you to review and provide feedback on the economic models (decision tree models) for PICC and UVC insertion provided in these slides.

- Following this slide, we have provided some background information to assist you with completing this exercise.

- It should take approximately 45 minutes to review these slides and provide feedback.

Let's begin!

Guide for navigating the decision tree

The decision tree diagram has branches which each represent a potential pathway or set of outcomes for a patient or group of patients.

The tree consists of the following:

1) Chance node: 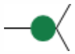 Where two alternative events for the patient is possible.

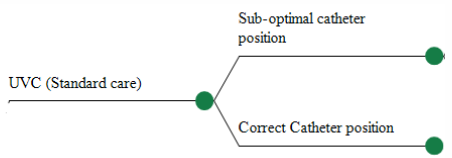

For example: A patient after catheter insertion can either have a 'sub-optimal catheter position' or 'correct catheter position'.

2) Pathways: A sequence of events moving from left to right through the tree.

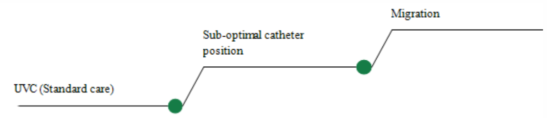

For example: A patient after insertion could move to a sub-optimal catheter position, then the catheter could migrate.

4) Terminal node: 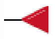 End of the patient pathway included in the model.

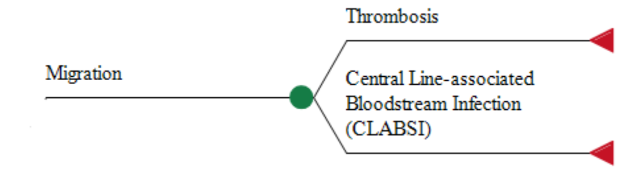

For example: In this scenario, a patient has had catheter migration and can experience and a complication (thrombosis or a CLABI).

Completion of patient journey

Just for the purpose of this work:

We follow a patient up until the removal of a successfully inserted catheter, removal could be due to the following:

1) Treatment completion; or
2) Removal due to a complication.

An insertion is considered successful once the device has been used for treatment.

Griffith UNIVERSITY
Centre for Applied Health Economics

Model confirmation

Peripherally Inserted Central Catheter (PICC) in Neonates

PICC model overview

- The next slide is an overview of a PICC insertion in a neonatal patient and potential pathways mapped out.
- The pathways after migration are not shown for clarity. But do expand out to complications associated with migration (shown as Asterix * on the next slide).
- After showing you the model overview, we will break the model down into smaller pieces. For each pathway we will seek your opinion on steps we may have missed, and the resources that are used within your clinical setting.

Griffith UNIVERSITY
Centre for Applied Health Economics

PICC insertion model (neonates)

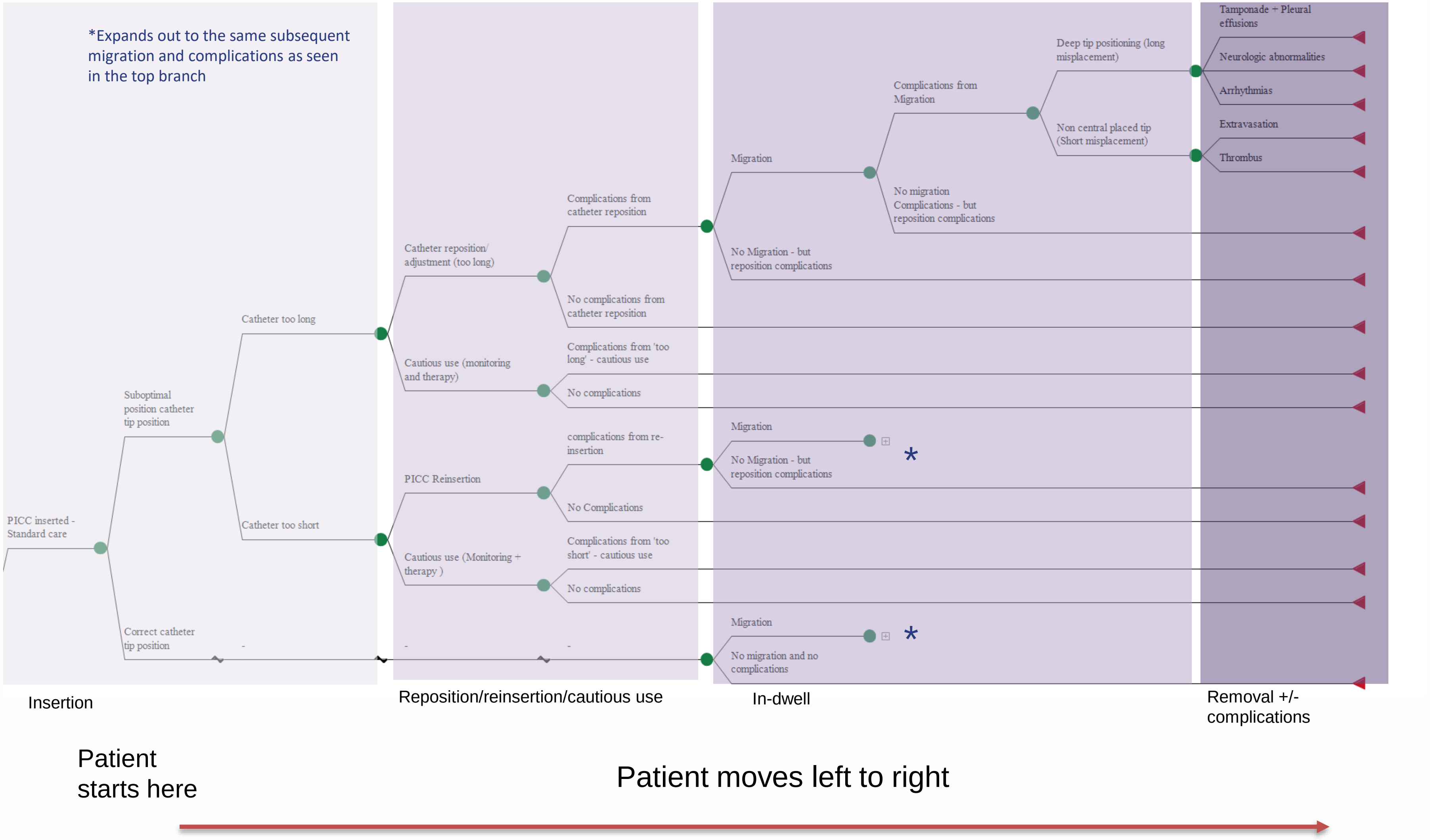

Potential patient pathways (PICC)

- The next slide shows potential pathways of a patient after catheter insertion.
- In our analysis: after insertion, a catheter tip would be in either the ‘correct position’ or ‘sub-optimal position’.
- After ‘sub-optimal position’, the catheter would be either inserted ‘too long’ or ‘too short. Catheter ‘too long’ can be repositioned or continue with ‘cautious use’. Catheter ‘too short’ would be reinserted or continue with ‘cautious use’.
- Reinsertion would only be with a PICC.
- Once a correct tip position has been achieved, the catheter could move towards migration and no migration.

Potential patient pathways

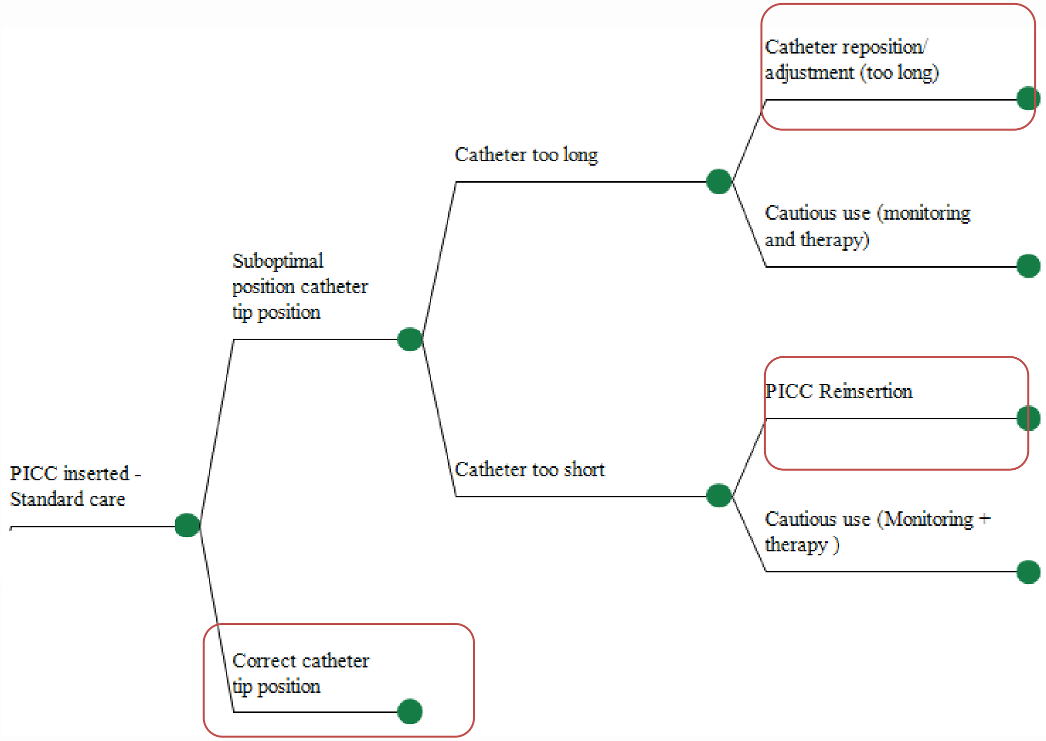

The highlighted boxes indicate the main potential pathways a patient may move into following catheter insertion.

These pathways and their subsequent complications from catheter tip migration will be our focus.

PICC model outcomes – Complications associated with migration

- The following slide shows how a catheter tip migration could result in complications or no complications.

- If there are complications, these are then separated into 'deep tip positioning' or 'non-central placed' tip related complications

Complications associated with catheter tip migration (in-dwell)

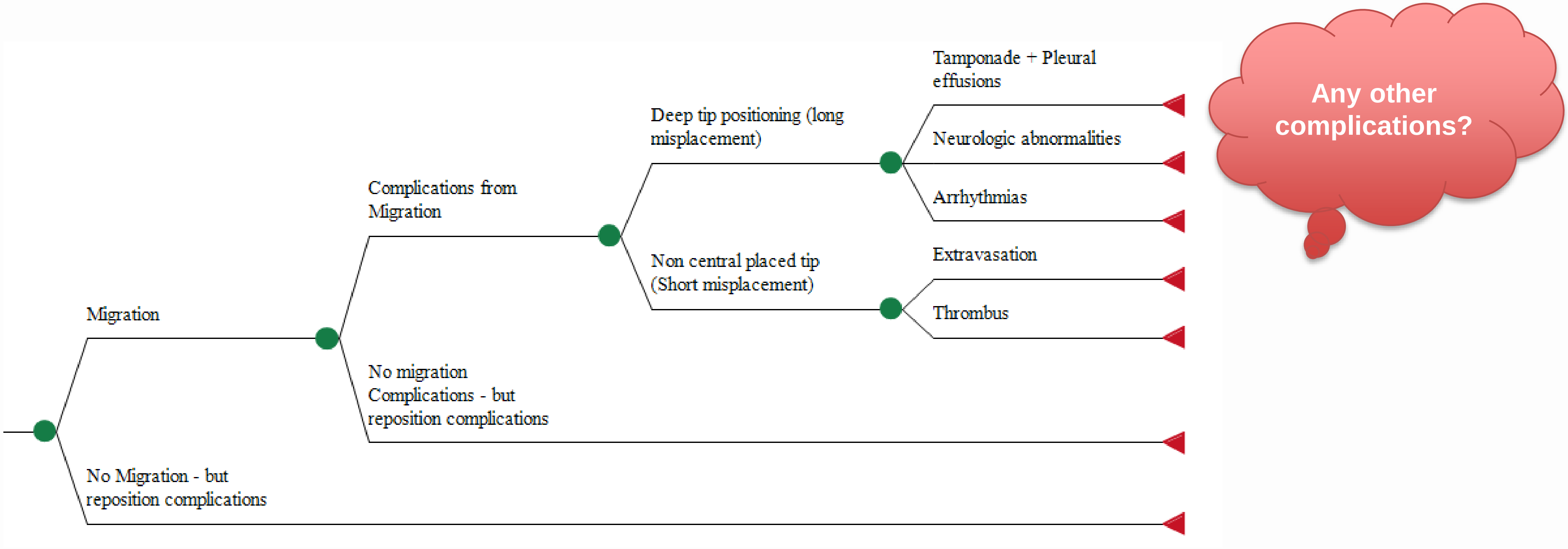

Are there any other complications associated with PICC tip migration?

Complications associated with migration		Comments
Cardiac Tamponade +/or pleural effusion		
Neurological complications		
Arrhythmias		
Extravasation		
Thrombus	CLABSI post-thrombus	
**Please specify other complications associated with migration**		**Comments**

Are there any other complications associated with catheter tip migration in-dwell?

?

[please enter your response here]

Pathways and resources

- For each pathway, we have identified the required resources for each step.
- We understand that this could be quite different to your clinical setting, so we seek your feedback on this topic.

Catheter (PICC) insertion and positioning – Standard care

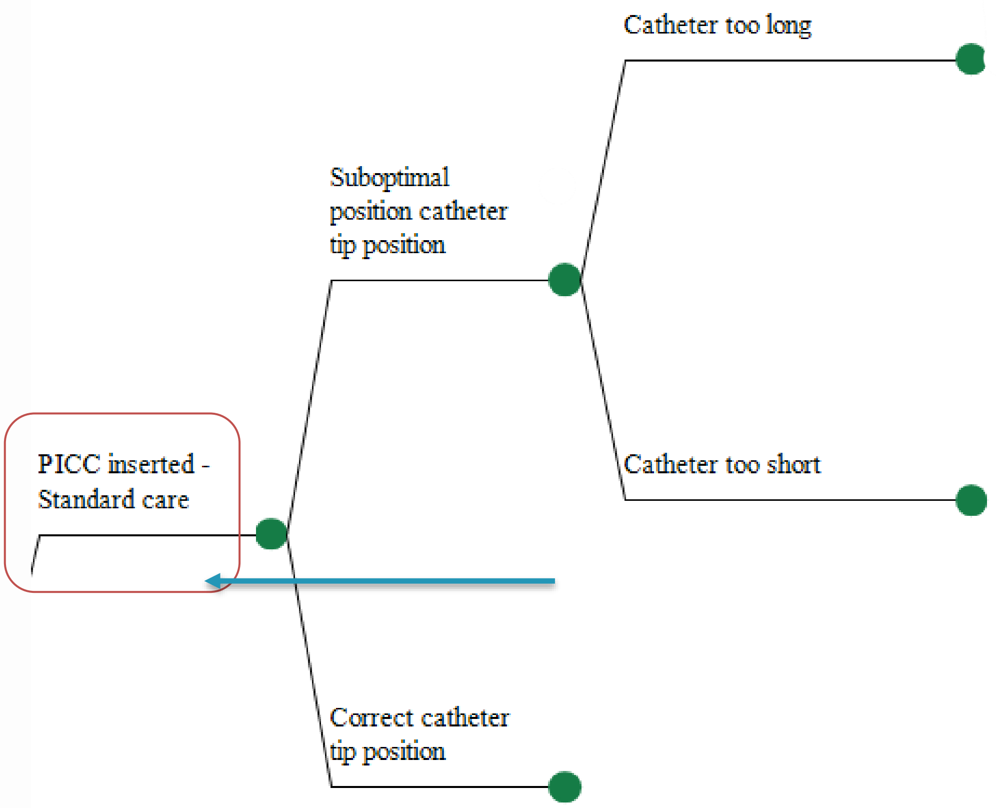

The table below shows our estimates of the resources used for PICC insertion – includes post insertion confirmation of catheter position (x-ray).

PICC insertion (Standard care) resource use		
**Time spent (minutes)**	Minutes	Units (if not time related)
Clinical nurse specialist	30	-
Nurse practitioner	30	-
PA (Physician's assistant)	30	-
Neonatologist	7.5	-
X-ray cost including radiologist and Radiographer fee	-	1
Device cost + consumables costs	-	1

Resource use
Considerations/questions?

For catheter (PICC) insertion and positioning – Standard care:

- In your clinical setting, what personnel are allocated during insertion of a PICC and for how long?
- What other resources are used in your clinical setting? (personnel + equipment)
- How many x-rays are taken after insertion to determine catheter position in your clinical setting?
- A table on the next slide will ask you for your best estimates.

Resource Use: Please complete

PICC insertion and positioning – Standard care

**PICC insertion**	**Our estimates:**		**Your estimates:**	
**Time spent (minutes)**	Minutes	Units (if not time related)	Minutes	Units (if not time related)
Registered nurse	-	-		
Clinical nurse specialist	30	-		
Nurse practitioner	30	-		
Resident	-	-		
PA (Physician's assistant)	30	-		
Neonatologist	7.5	-		
Other non-nursing and non-medical specialists	-	-		
X-ray cost including radiologist and Radiographer fee	-	1		
Device and consumables (see next page for details)	-	1		
Other resources required	-	-		
Please specify and add rows as needed	-	-		

Resource use: Device and consumables for PICC insertion

- Original PICC Placement Kit or Guided PICC placement kit
- Chest X-ray
- PICC Placement and repositioning with with Fluoroscopic Guidance
- Gloves, mask and gown
- Catheter device plus cannula
- Chlorhexidine Acetate Aq Soln 0.05% w/v

Question – Are there any other consumables or equipment used in your setting?

[please enter your response here]

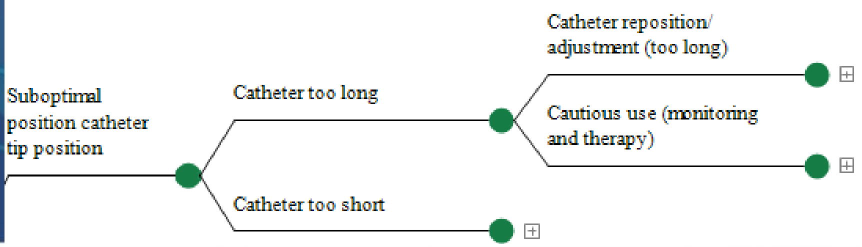

Catheter too long (PICC) - Requiring catheter reposition

The following slide shows the patient pathway of sub-optimal positioned catheter →inserted too long →catheter reposition.

Assumptions in this arm:

- X-ray/radiography would be conducted to determine catheter length.
- ‘Catheter too long’ requires another ‘monitoring phase’ (including x-ray)
- ‘Catheter too long’ requires the catheter to be repositioned (including x-ray to confirm positioning).

Catheter too long (PICC) - requiring catheter reposition

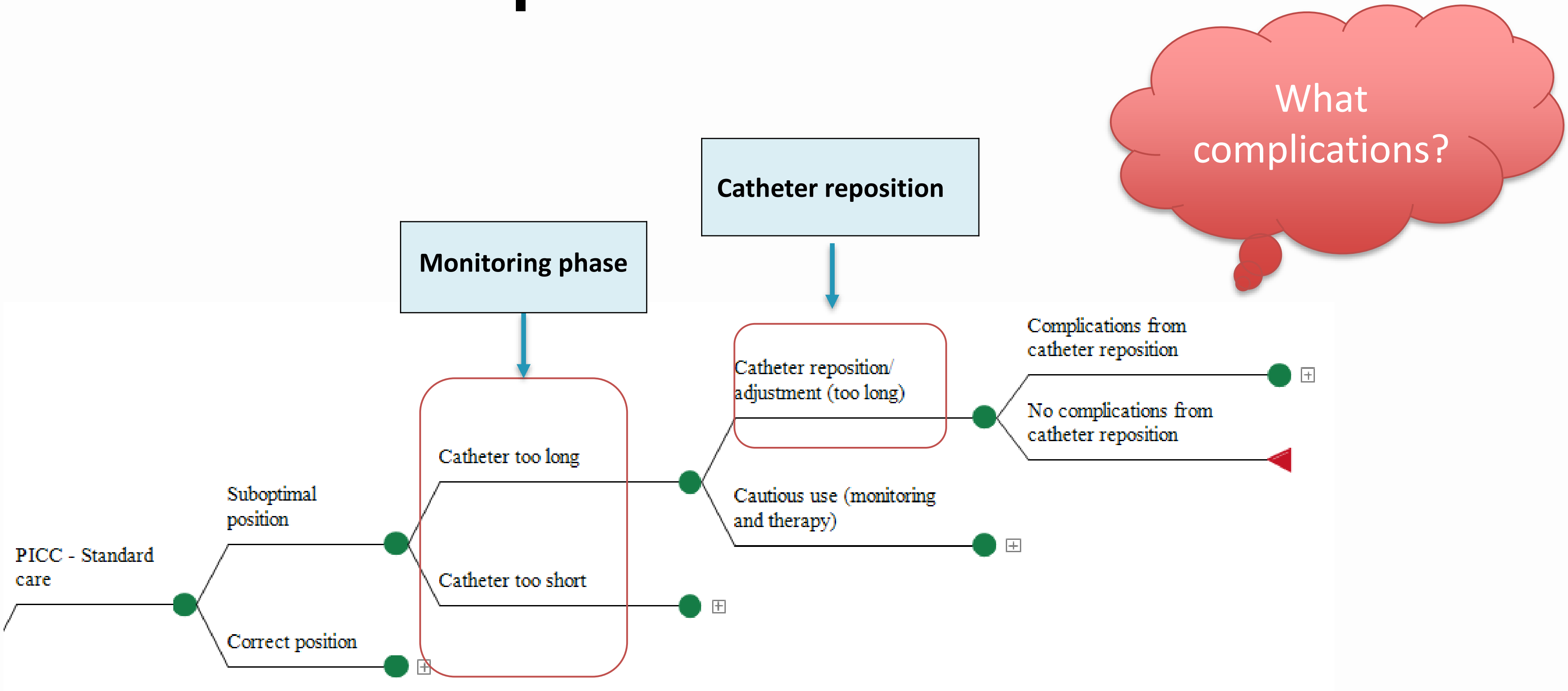

Complications

- We have identified increased risk of air embolism and or CLABSIs as a complication for reinsertion.

?

Question – Are there any other complications associated with catheter reposition (PICC)?

[please enter your response here]

Resource use
Considerations/questions?

For catheter too long (PICC) – Requiring catheter reposition:

- Are our resource use estimates correct?
- What other resources are used in your clinical setting? (personnel + equipment)
- Two tables on the following slides will ask you for your best estimates.

Resource Use - Please Complete

<u>Monitoring phase</u>: registered nurse, resident and an x-ray to confirm position

**Monitoring phase**	**Our estimates:**		**Your estimates:**	
**Time spent (minutes)**	Minutes	Units	Minutes	Units (if not time related)
Registered nurse	15	-		
Clinical nurse specialist	-	-		
Nurse practitioner		-		
Resident	15	-		
PA (Physician's assistant)	-	-		
Neonatologist	-	-		
Other non-nursing and non-medical specialists	-	-		
X-ray cost including radiologist and Radiographer fee	-	1		
device cost + consumables costs	-	-		
Other resources required	-	-		
Please specify and add rows as needed	-	-		

Griffith UNIVERSITY
Centre for Applied Health Economics

Resource use - Please complete

<u>Catheter reposition</u>: registered nurse, resident, neonatologist and an x-ray to confirm position.

**Catheter reposition**	**Our estimates**		**Your estimates:**	
**Time spent (minutes)**	Minutes	Units	Minutes	Units (if not time related)
Registered nurse	-	-		
Clinical nurse specialist	15	-		
Nurse practitioner	-	-		
Resident	15	-		
PA (Physician's assistant)	-	-		
Neonatologist	5	-		
Other non-nursing and non-medical specialists	-	-		
X-ray cost including radiologist and Radiographer fee	-	1		
device cost + consumables costs	-	-		
Other resources required	-	-		
Please specify and add rows as needed				

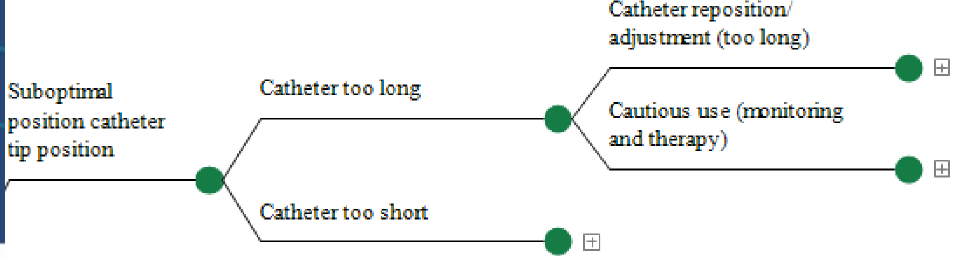

Catheter too long (PICC) - Cautious use

The following slide shows the patient pathway of ‘sub-optimal positioned catheter →inserted too long. However, catheter is left in and ‘cautious use’

Main assumptions in this arm:

- X-ray/radiography would be conducted to determine catheter length;
- A clinical decision has been made continue using the long catheter.
- ‘Cautious use’ does not require another x-ray, however it could require further therapy and monitoring.

Catheter too long (UVC) - Cautious use

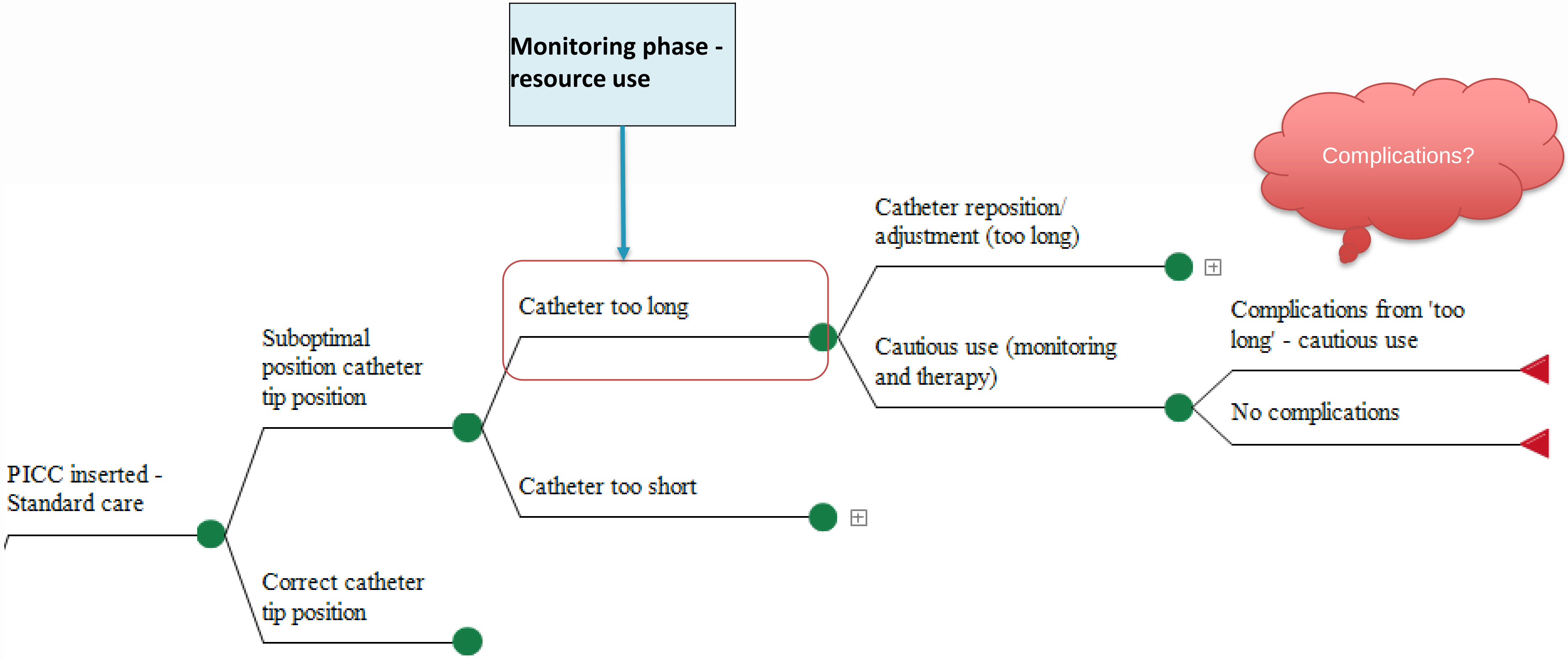

Have we missed any complications associated with catheter too long (PICC) – Cautious use?

?

[please enter your response here]

Resource use
Considerations/questions?

For catheter too long (PICC) – Cautious use:

- Are our resource use estimates correct?
- What other resources are used in your clinical setting? (personnel + equipment)
- A table on the next slide will ask you for your best estimates.

Resource use - Please complete

Cautious use (Monitoring and therapy – too long): Registered nurse, nurse practitioner and a physician

Cautious use (monitoring and therapy)	Our estimates		Your estimates:	
**Time spent (minutes)**	Minutes	Units	Minutes	Units (if not time related)
Registered nurse	30	-		
Clinical nurse specialist	-	-		
Nurse practitioner	30	-		
Resident	-	-		
PA (Physician's assistant)	-	-		
Neonatologist	15	-		
Other non-nursing and non-medical specialists	-	-		
X-ray cost including radiologist and Radiographer fee	-	-		
UVC device cost + consumables costs	-	-		
Other resources required (Extra IV resources required?)	-	-		
Please specify and add rows as needed	-	-		

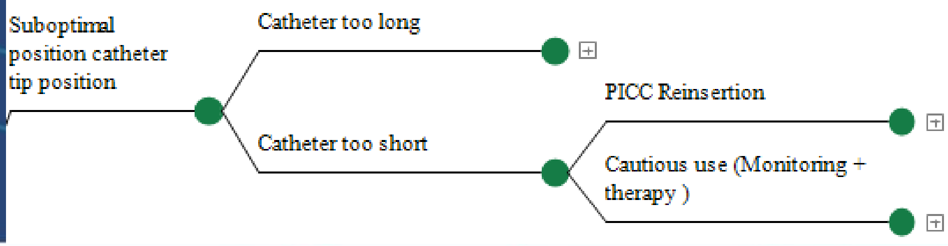

Catheter too short (PICC) – Reinsertion

The following slide shows the patient pathway of ‘sub-optimal positioned catheter →inserted too short →catheter reinsertion.

Main assumptions in this arm:

- Catheter too short does not get repositioned, instead a new catheter is required.
- An x-ray would be required following reinsertion to determine catheter length
- A new catheter inserted would also be a PICC.

The main complication identified with reinsertion is increased risk of air embolism and or CLABSIs.

Catheter too short - PICC reinsertion

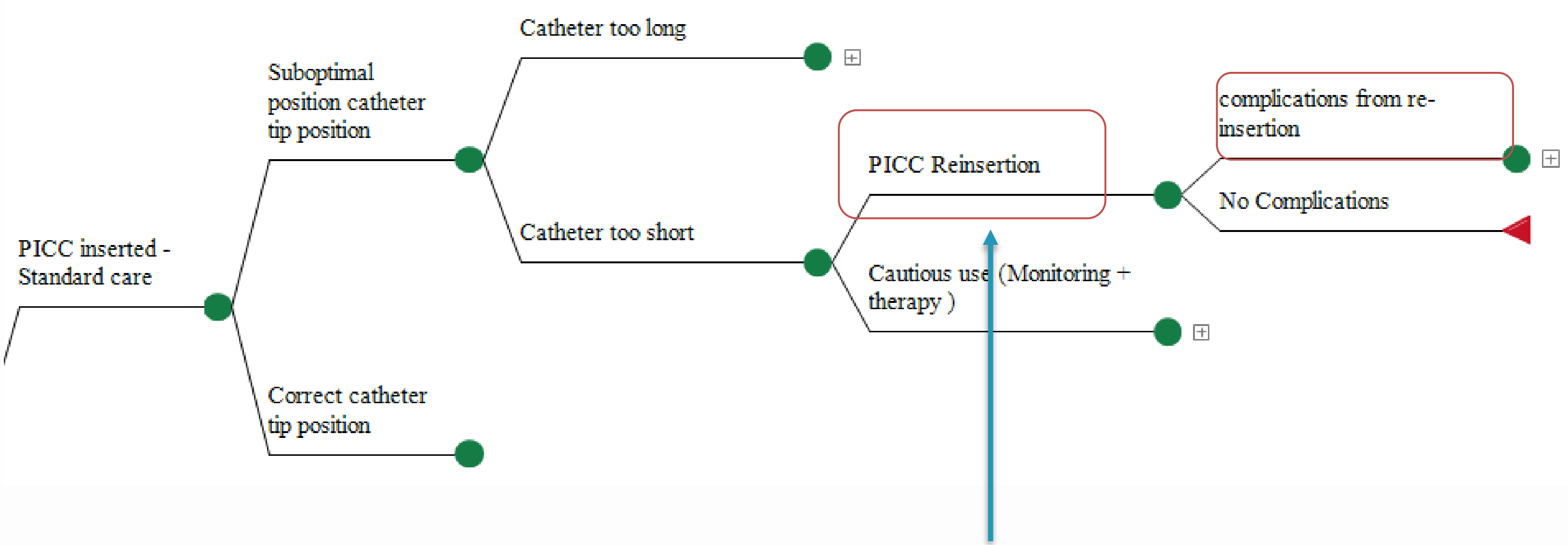

PICC (removal + reinsertion)	
Procedures required:	Units:
Catheter removal	1
Catheter insertion	1
Extra resources?	?

Resource use: Device and consumables for PICC reinsertion

- Original PICC Placement Kit
- Guided PICC Placement Kit
- Chest X-ray
- PICC Placement with Fluoroscopic Guidance
- PICC Repositioning with Fluoroscopic Guidance
- Catheter device

?

Question – Are there any other consumables or equipment used in your setting?

[please enter your response here]

Griffith UNIVERSITY
Centre for Applied Health Economics

Have we missed any complications associated with catheter too short (PICC) – Reinsertion?

?

[please enter your response here]

Catheter too short (PICC) - Cautious use

The following slide shows the patient pathway of ‘sub-optimal positioned catheter →inserted too short →cautious use.

Main assumptions in this arm:

- Catheter too short does not get repositioned, instead a clinical decision has been made to keep using the short catheter.
- Complications from this ‘too short’ would result in catheter withdrawal.
- Catheter too short requires another monitoring phase (including x-ray)
- Cautious use requires another monitoring phase (including x-ray)

Catheter too short (PICC) - Cautious use

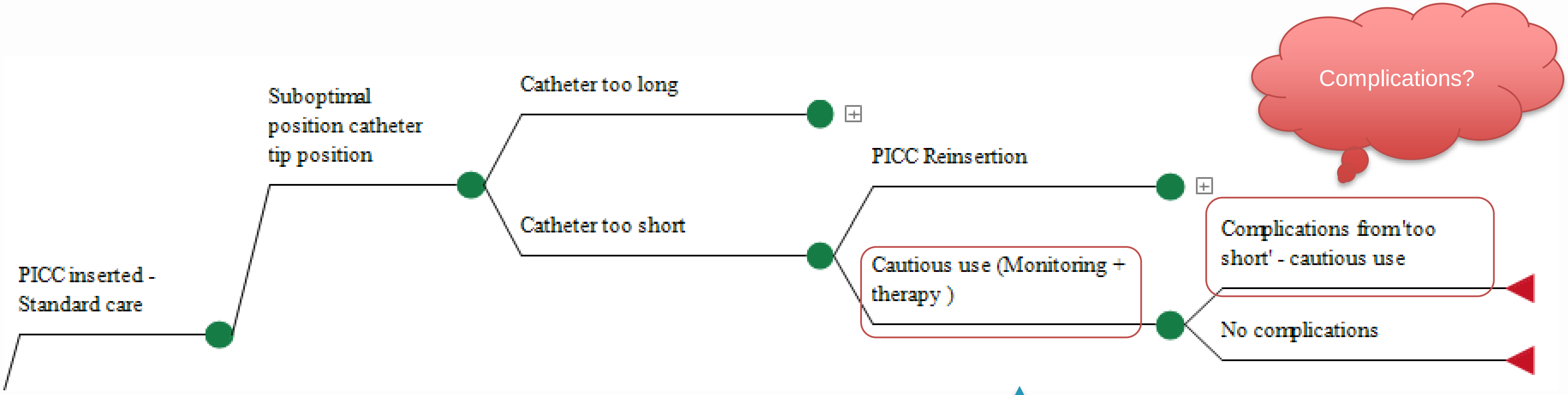

Cautious use (monitoring and therapy) – resource use

Have we missed any complications associated with catheter too short (PICC) – Cautious use?

?

[please enter your response here]

Resource use
Considerations/questions?

For catheter too short (PICC) – Cautious use:

- Are our resource use estimates correct?
- What other resources are used in your clinical setting? (personnel + equipment)
- A table on the next slide will ask you for your best estimates.

Resource use – Please Complete

Cautious use (Monitoring and therapy - too short): Registered nurse, nurse practitioner and a physician

**Cautious use (monitoring and therapy)**	**Our estimates**		**Your estimates:**	
**Time spent (minutes)**	Minutes	Units	Minutes	Units (if not time related)
Registered nurse	30	-		
Clinical nurse specialist	-	-		
Nurse practitioner	30			
Resident	-	-		
PA (Physician's assistant)		-		
Neonatologist	15	-		
Other non-nursing and non-medical specialists	-	-		
X-ray cost including radiologist and Radiographer fee	-	1		
UVC device cost + consumables costs	-	-		
Other resources required (Extra IV resources required?)	-	-		
Please specify and add rows as needed				

Any general comments about the PICC model?

?

[please enter your response here]

Griffith UNIVERSITY
Centre for Applied Health Economics

Model confirmation

Umbilical Venous Catheter (UVC) in Neonates

UVC model overview

- The next slide is an overview of an UVC insertion in a neonatal patient and potential pathways mapped out.
- The pathways after migration are not shown for clarity. But do expand out to complications associated with migration (shown as Asterix * on the next slide).
- After showing you the overview, we will break down the model into smaller pieces and seek your opinion. For each pathway we will seek your opinion on steps we may have missed, and the resources that are used within your clinical setting.

GriffithUNIVERSITY
Centre for Applied Health Economics

UVC insertion model (neonates)

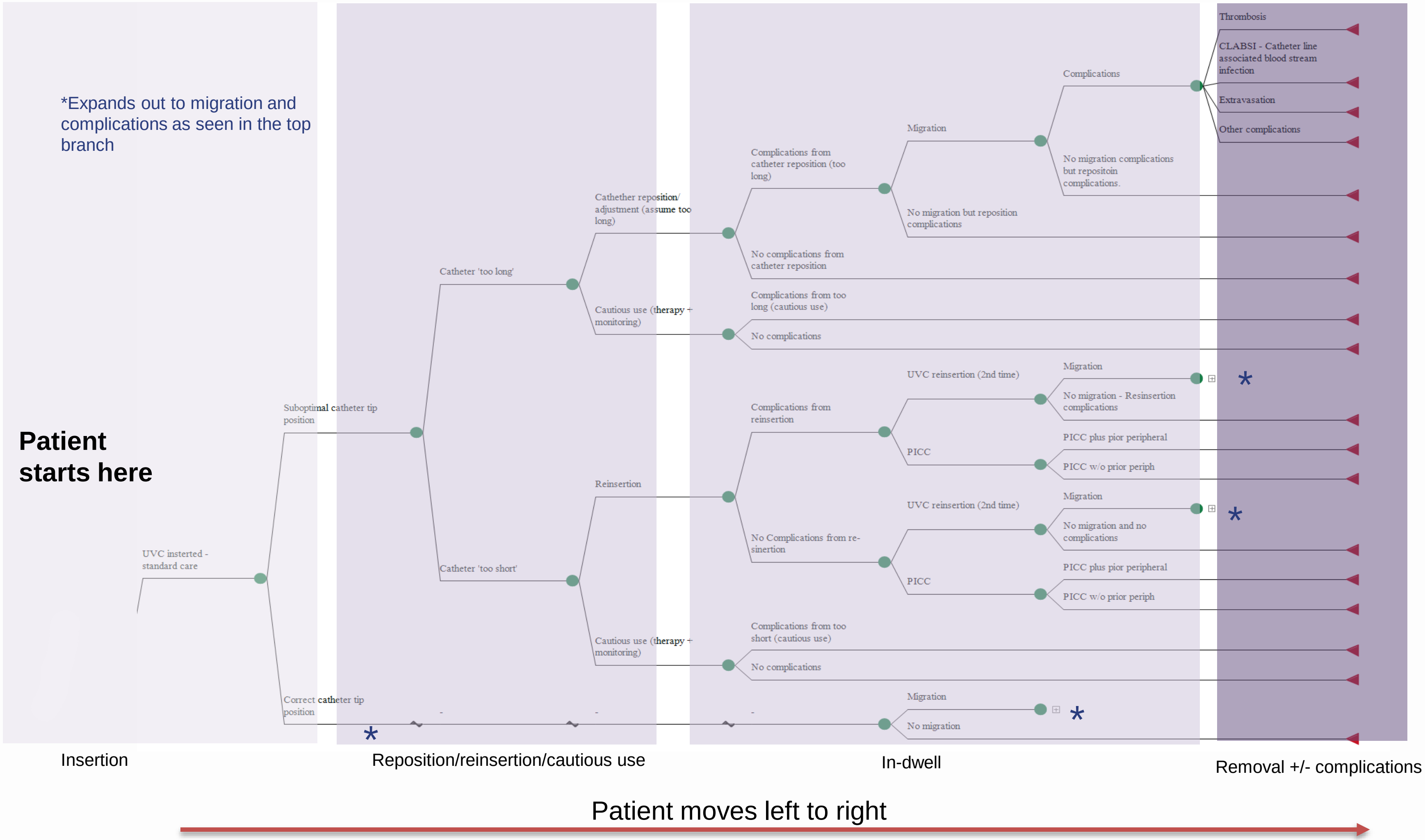

Potential patient pathways (UVC)

- The next slide shows potential pathways of a patient after catheter insertion.
- In our analysis: after insertion, a catheter tip would be in either the ‘correct position’ or ‘sub-optimal position’.
- After ‘sub-optimal position’, the catheter would be either inserted ‘too long’ or ‘too short. Catheter ‘too long’ can be repositioned or continue with ‘cautious use’. Catheter ‘too short’ would be reinserted or continue with ‘cautious use’.
- Reinsertion in this case could either be UVC or PICC.
- Once a correct tip position has been achieved, the catheter could move towards migration and no migration.

Potential patient pathways

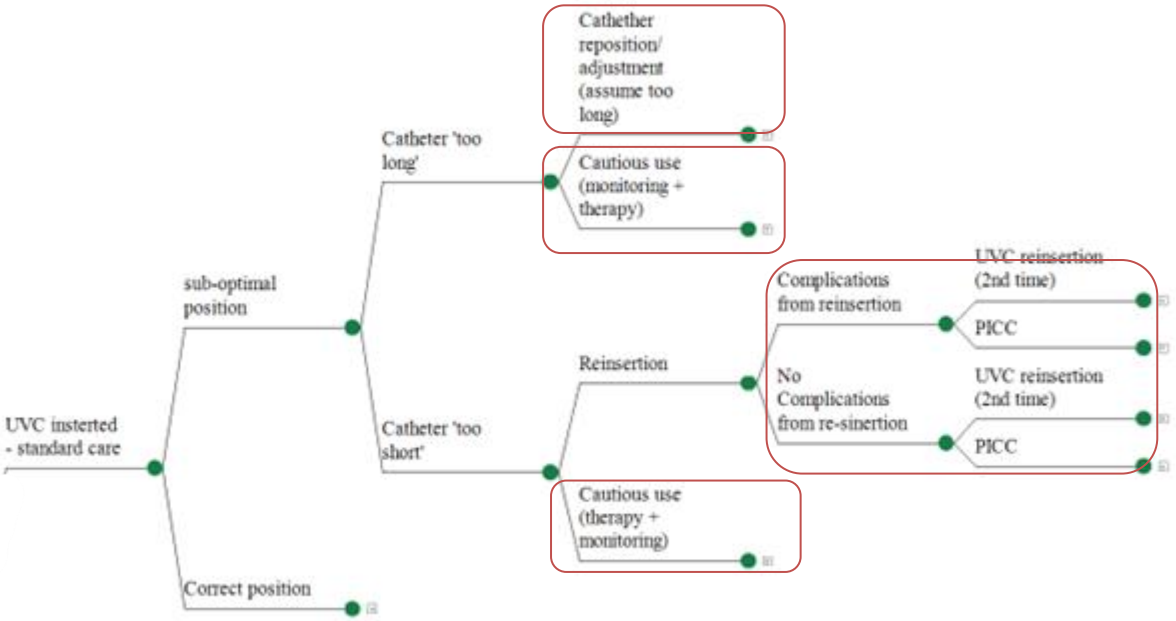

The highlighted boxes indicate the main potential pathways a patient may into following catheter insertion.

These pathways and their subsequent complications from catheter tip migration will be our focus.

UVC model outcomes - Complications associated with migration

- The following slide shows how a catheter tip migration could result in complications or no complications.

- Complications include thrombosis, CLABSI's, extravasation and other complications.

Further detail of two complications (Extravasation and other complications):

- Extravasation includes Pericardial + pleural effusion, cardiac tamponade, hepatic extravasation.

- Other complications could include: necrotising enterocolitis, intra-abdominal extravasation, and endocarditis.

Migration and complications

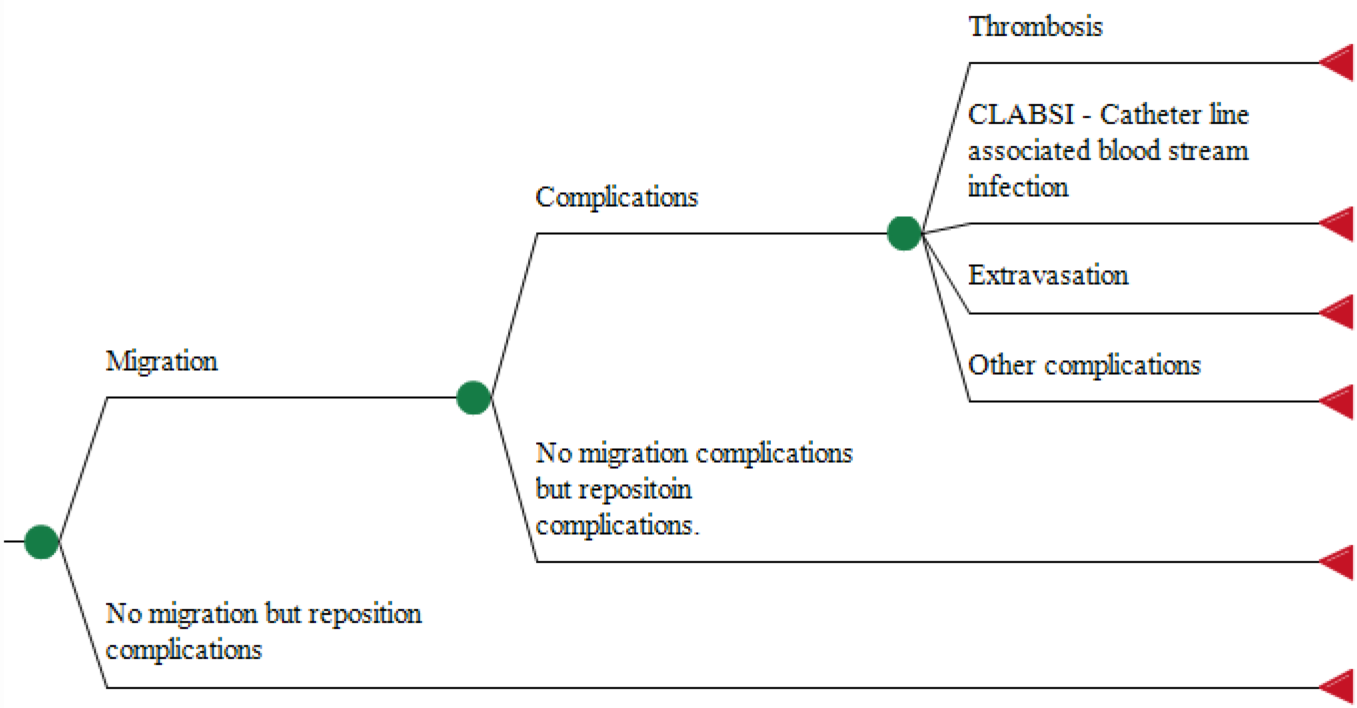

Are there any other complications associated with UVC tip migration?

Complications associated with migration		Comments
Thrombosis		
CLABSI		
Extravasation	Hepatic extravasation, intra-abdominal extravasation, cardiac tamponade, and pleural effusion.	
Other complications	necrotising enterocolitis, and endocarditis	
**Please specify other complications associated with migration**		**Comments**

Catheter insertion (UVC) and positioning – Standard care

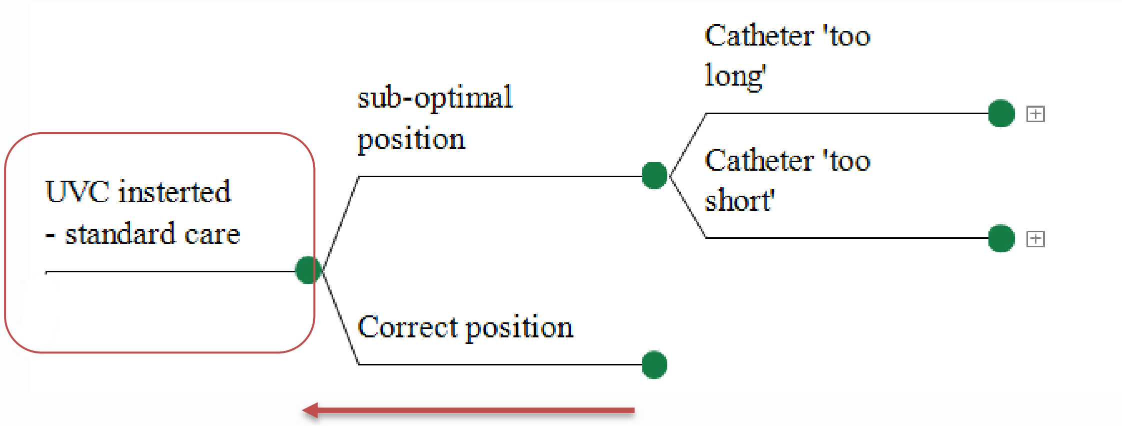

The table below shows our estimates of the resources used for UVC insertion – includes post insertion confirmation of catheter position (x-ray).

UVC insertion (Standard care) resource use		
**Time spent (minutes)**	Minutes	Units (if not time related)
Clinical nurse specialist	30	-
Nurse practitioner	30	-
Resident	15	-
PA (Physician's assistant)	-	-
Neonatologist	10	-
X-ray cost including radiologist and Radiographer fee	-	1
Device cost + consumables costs	-	1

Resource use
Considerations/questions?

- In your clinical setting, what personnel are allocated during insertion of a UVC and for how long?
- What other resources are used in your clinical setting? (personnel + equipment)
- How many x-rays are taken after insertion to determine catheter position in your clinical setting?
- A table on the next slide will ask you for your best estimates.

Resource use – Please Complete

Catheter insertion (UVC) and positioning – Standard care

**UVC insertion**	**Our estimates:**		**Your estimates:**	
**Time spent (minutes)**	Minutes	Units (if not time related)	Minutes	Units (if not time related)
Registered nurse	-	-		
Clinical nurse specialist	30	-		
Nurse practitioner	30	-		
Resident	15	-		
PA (Physician's assistant)	-	-		
Neonatologist	10	-		
Other non-nursing and non-medical specialists	-	-		
X-ray cost including radiologist and Radiographer fee	-	1		
Device and consumables	-	1		
Other resources required	-	-		
Please specify and add rows as needed				

Resource use :Device and consumables for UVC insertion

- Original PICC Placement Kit
- Guided PICC Placement Kit
- Chest X-ray
- Umbilical catheter
- Instrument pack
- Sterile gloves and gown
- 3-way taps and syringes with NaCl
- 2 % aq. chlorhexidine soln as skin prep

?

Question – Are there any other consumables or equipment used in your setting?

[please enter your response here]

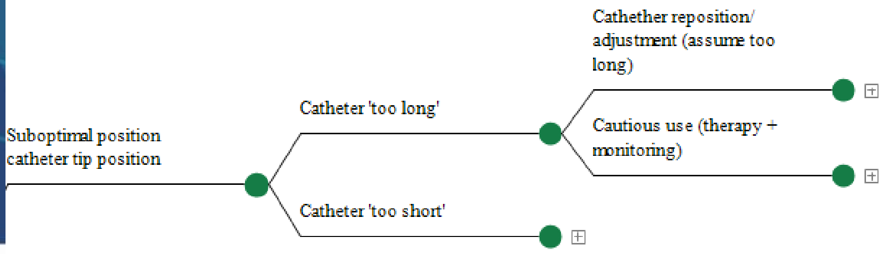

Catheter too long (UVC) - Requiring catheter reposition

The following slide shows the patient pathway of ‘sub-optimal positioned catheter →inserted too long →catheter reposition.

Assumptions in this arm:

- X-ray/radiography would be conducted to determine catheter length.
- Catheter too long would require the catheter to be repositioned and subsequent x-ray to confirm positioning.

Catheter too long (UVC) - Requiring catheter reposition

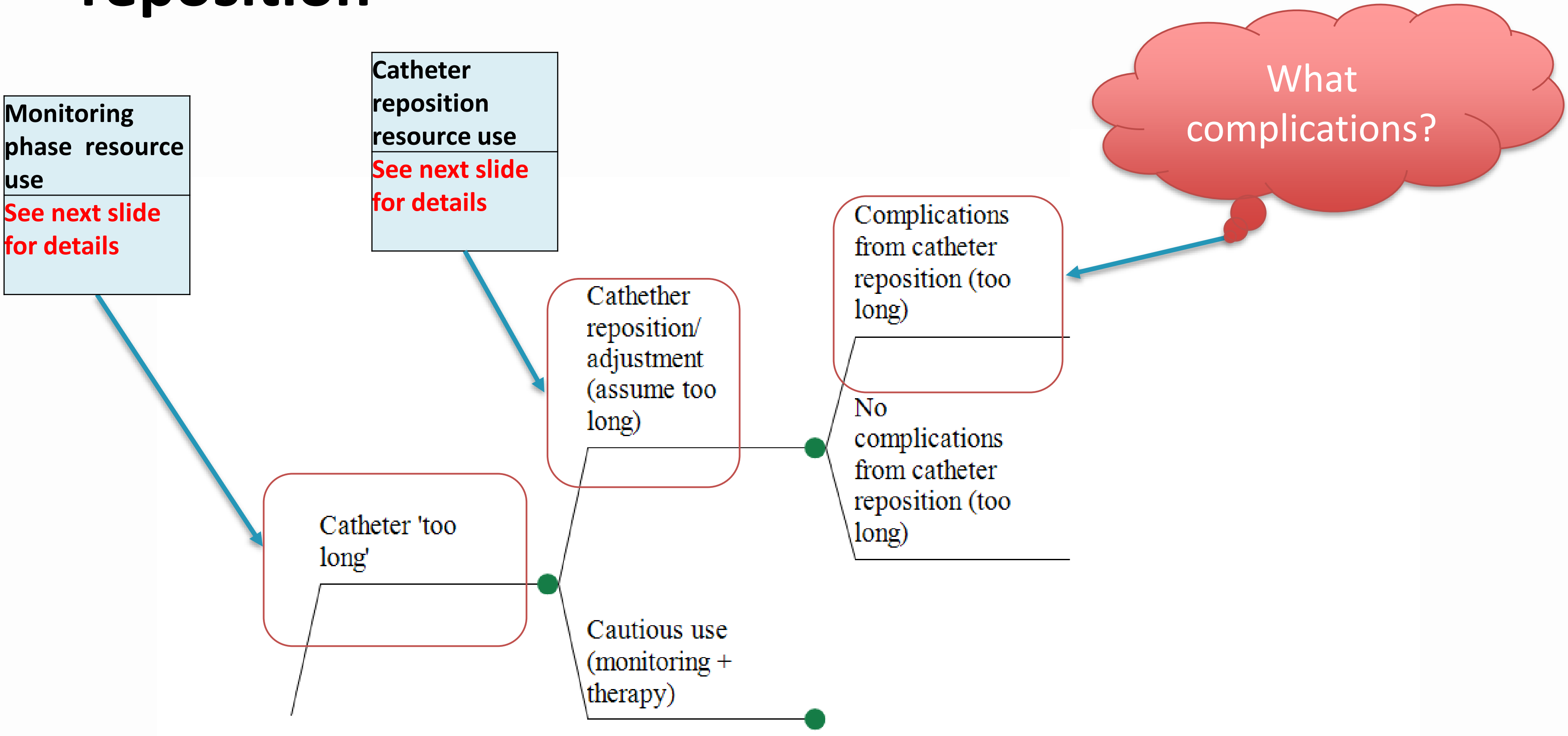

Complications

- We have identified increased risk of CLABSIs as a complication associated with reinsertion.

?

Question – Are there any other complications associated with catheter reposition (UVC)?

[please enter your response here]

Resource use
Considerations/questions?

For catheter too long (UVC) – Requiring catheter reposition:

- Are our resource estimates correct?
- What other resources are used in your clinical setting? (personnel + equipment)
- Two tables on the following slides will ask you for your best estimates.

Resource use - Please complete

Monitoring phase (UVC) - registered nurse, resident and an x-ray to confirm position

**Monitoring phase**	**Our estimates**		**Your estimates:**	
**Time spent (minutes)**	Minutes	Units	Minutes	Units (if not time related)
Registered nurse	15	-		
Clinical nurse specialist	-	-		
Nurse practitioner	-	-		
Resident	15	-		
PA (Physician’s assistant)	-	-		
Neonatologist	-	-		
Other non-nursing and non-medical specialists	-	-		
X-ray cost including radiologist and Radiographer fee	-	1		
UVC device cost + consumables costs	-	-		
Other resources required	-	-		
Please specify and add rows as needed				

Griffith UNIVERSITY
Centre for Applied Health Economics

Resource use - Please complete

Catheter reposition (UVC) - registered nurse, resident, neonatologist and an x-ray to confirm position.

**Catheter reposition**	**Our estimates**		**Your estimates:**	
**Time spent (minutes)**	Minutes	Units	Minutes	Units (if not time related)
Registered nurse	15	-		
Clinical nurse specialist	15	-		
Nurse practitioner		-		
Resident	15	-		
PA (Physician's assistant)	-	-		
Neonatologist	5	-		
Other non-nursing and non-medical specialists	-	-		
X-ray cost including radiologist and Radiographer fee	-	1		
UVC device cost + consumables costs	-	-		
Other resources required	-	-		
Please specify and add rows as needed				

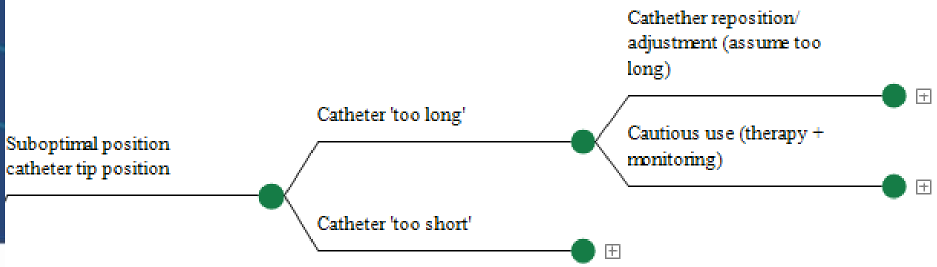

Catheter too long (UVC) - Cautious use

The following slide shows the patient pathway of ‘sub-optimal positioned catheter →inserted too long. However, the catheter continues to be used and is deemed ‘cautious use’.

Main assumptions in this arm:

- X-ray/radiography would be conducted to determine catheter length.
- A clinical decision has been made continue using the long catheter.
- ‘Cautious use’ does not require another x-ray, however it could require further therapy and monitoring.

Catheter too long (UVC) - Cautious use

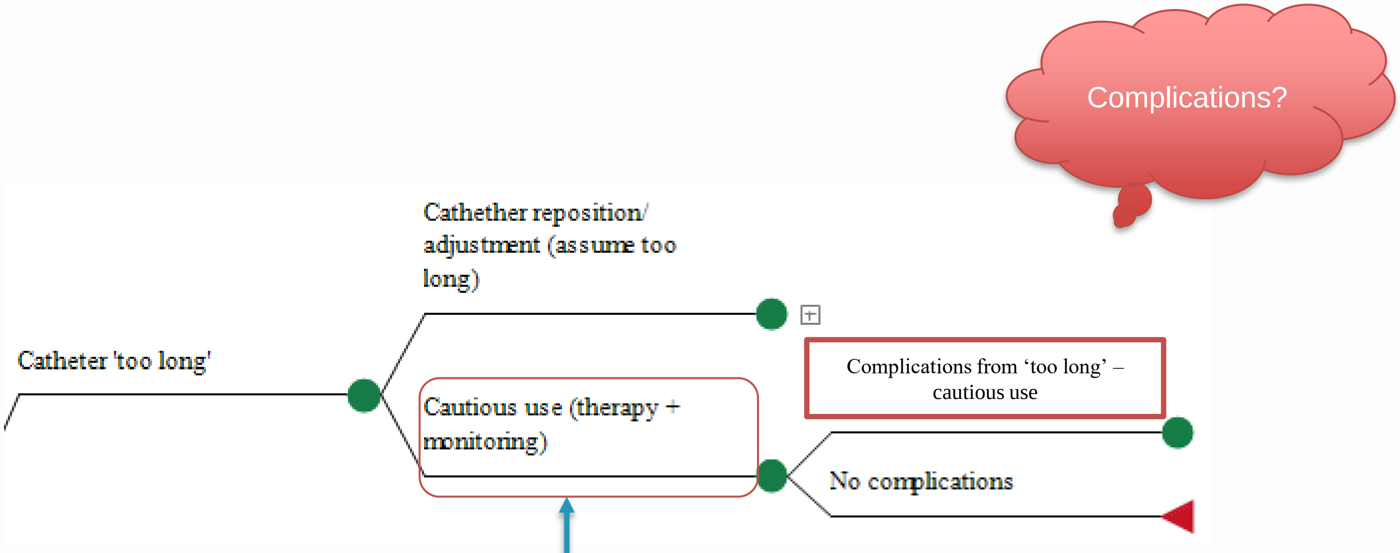

Cautious use (therapy + monitoring) - resource use

See next slide for details

What are the complications associated with continuing to use a catheter which has been inserted too long (UVC) – Cautious use?

?

[please enter your response here]

Resource use
Considerations/questions?

For catheter too long (UVC) – Cautious use:

- Are our resource use estimates correct?
- What other resources are used in your clinical setting? (personnel + equipment)
- A table on the next slide will ask you for your best estimates.

Griffith UNIVERSITY
Centre for Applied Health Economics

Resource use – Please complete

Cautious use (Monitoring and therapy – too long): Registered nurse, nurse practitioner and a physician

Cautious use (monitoring and therapy)	Our estimates		Your estimates:	
**Time spent (minutes)**	Minutes	Units	Minutes	Units (if not time related)
Registered nurse	30	-		
Clinical nurse specialist	-	-		
Nurse practitioner	30	-		
Resident	-	-		
PA (Physician's assistant)	-	-		
Neonatologist	15	-		
Other non-nursing and non-medical specialists	-	-		
X-ray cost including radiologist and Radiographer fee	-	-		
UVC device cost + consumables costs	-	-		
Other resources required (Extra IV resources required?)	-	-		
Please specify and add rows as needed	-	-		

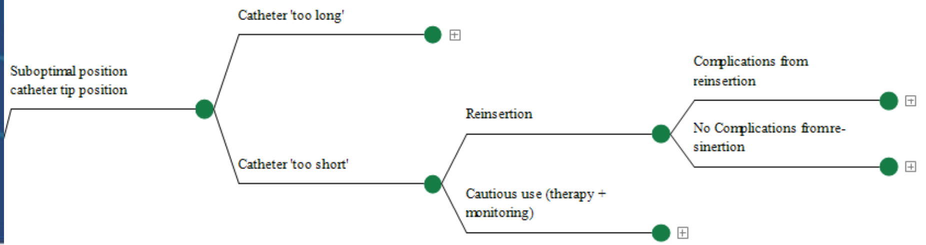

Catheter too short (UVC) - Reinsertion

The following slide shows the patient pathway of ‘sub-optimal positioned catheter →inserted too short →catheter reinsertion.

Main assumptions in this arm:

- Catheter too short does not get repositioned. A new catheter is required.
- The probability of using a UVC or PICC as the second insertion device is 50:50.

The main complication identified with reinsertion is increased risk of thrombosis and or CLABSIs.

Catheter too short - UVC/PICC reinsertion

2nd UVC insertion assumes correct catheter tip insertion but can still migrate

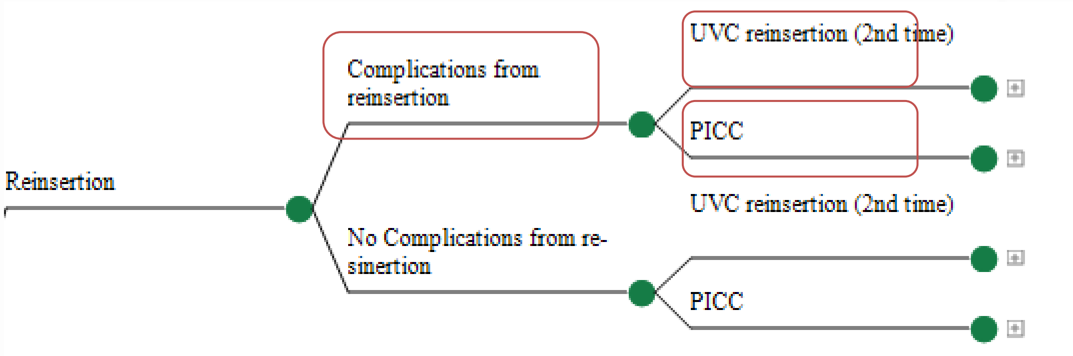

UVC removal +PICC insertion	
Procedures required:	Units:
Catheter removal	1
Catheter insertion	1
Extra resources?	?

Have we missed any complications associated with catheter too short– UVC/PICC insertion?

?

Complications associated with reinsertion	Comments
CLABSI	
Air embolism	
**Please specify other complications associated with reinsertion**	**Comments**

Resource use
Considerations/questions?

For catheter too short – UVC/PICC Reinsertion:

- Are our resource use estimates correct?
- What other resources are used in your clinical setting? (personnel + equipment)
- A table on the next slide will ask you for your best estimates.

Resource use – Please complete

Catheter too short - UVC/PICC reinsertion

**Cautious use (monitoring and therapy)**	**Our estimates**		**Your estimates:**	
**Time spent (minutes)**	Minutes	Units	Minutes	Units (if not time related)
Catheter removal	-	1		
UVC insertion (described earlier)	-	1		
Other resources required (Extra IV resources required?)	-	-		
Please specify and add rows as needed	-	-		

?

Question: What is the probability of using a UVC vs PICC after failure with UVC in your clinical setting?

[please enter your response here]

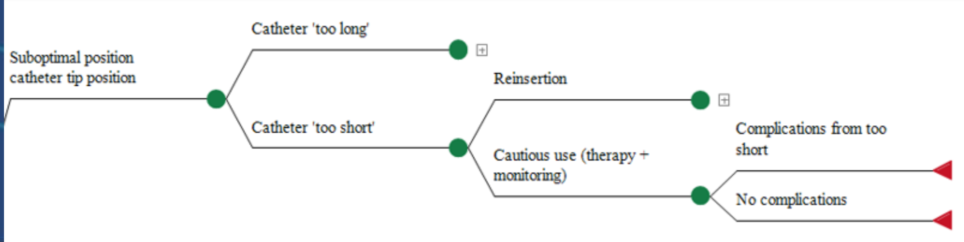

Catheter too short (UVC) - Cautious use

The following slide shows the patient pathway of ‘sub-optimal positioned catheter →inserted too short →cautious use

Main assumptions in this arm:

- Catheter inserted ‘too short’ would require an x-ray to confirm position.
- A clinical decision has been made continue using the short catheter.
- Cautious use’ does not require another x-ray, however it could require further therapy and monitoring.

Griffith UNIVERSITY
Centre for Applied Health Economics

Catheter too short (UVC) - Cautious use

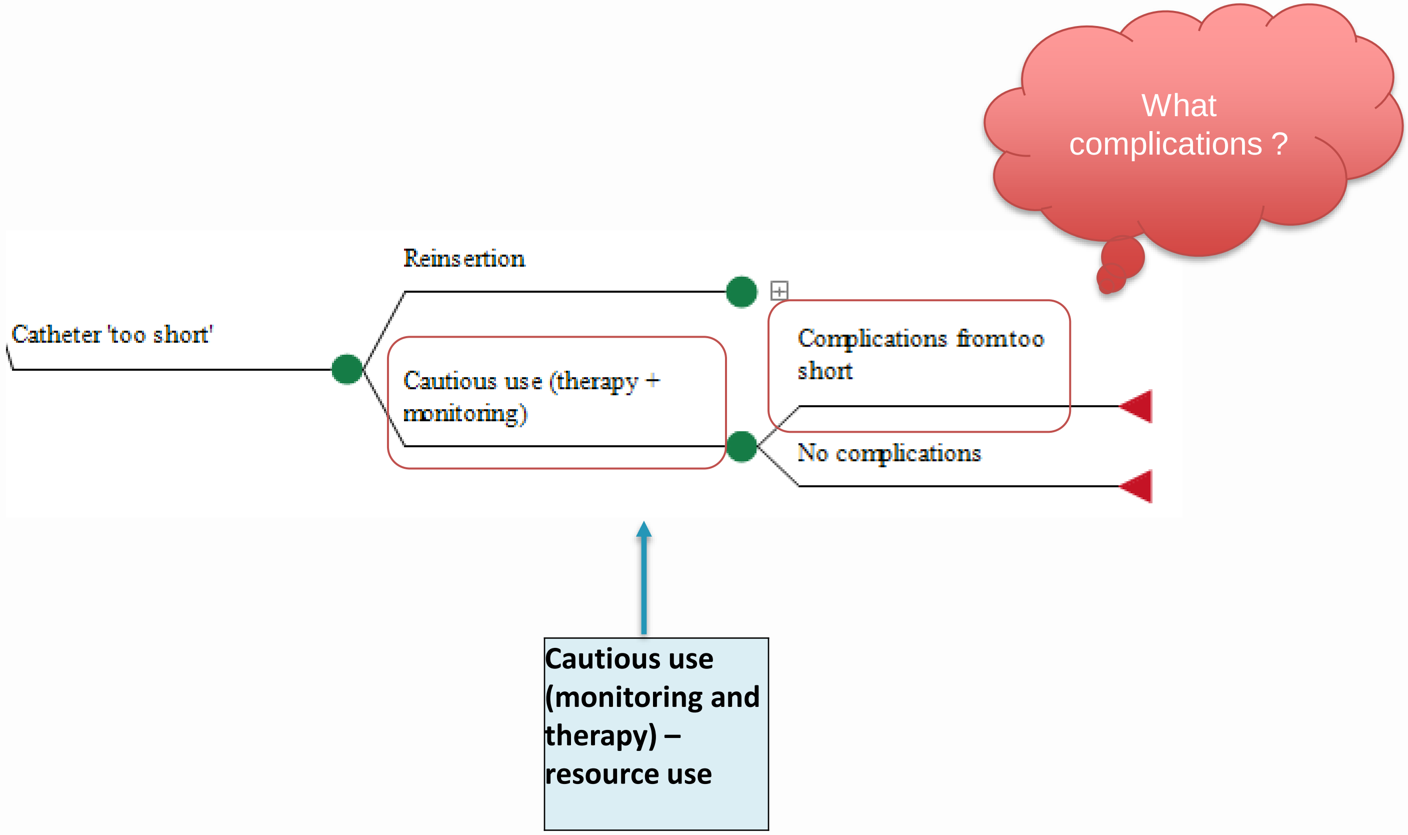

Resource use – Please complete

Cautious use (Monitoring and therapy – too short): Registered nurse, nurse practitioner and a physician/resident

**Cautious use (monitoring and therapy)**	**Our estimates**		**Your estimates:**	
**Time spent (minutes)**	Minutes	Units	Minutes	Units (if not time related)
Registered nurse	30	-		
Clinical nurse specialist	-	-		
Nurse practitioner	30	-		
Resident	30	-		
PA (Physician's assistant)	-	-		
Neonatologist	15	-		
Other non-nursing and non-medical specialists	-	-		
X-ray cost including radiologist and Radiographer fee	-	-		
UVC device cost + consumables costs	-	-		
Other resources required (Extra IV resources required?)	-	-		
Please specify and add rows as needed				

What complications are associated with using a 'too short' catheter in UVC setting?

?

[please enter your response here]

- Please specify other complications associated with reinsertion
- Comments

End of Activity

Thank you for your time completing this pre-focus group activity.

We look forward to meeting with you at the focus group session on the 14th October.
