# Supplementary material for: Umbilical venous catheter and peripherally inserted central catheter malposition and tip migration in neonates: A mixed methods cost analysis
Source: Int J Nurs Stud Adv. 2025 Nov 10;9:100450. doi: 10.1016/j.ijnsa.2025.100450 (PMC12664355; doi:10.1016/j.ijnsa.2025.100450)
Supplement: Supplementary file 2 [file mmc2.docx]

Table S1: Inputs extracted from purposive literature review

| **Model** | **Informed input parameter** | **Parameter Value (base case)** | **Source** | **Input Type** | **Study Type** | **Sample size** | **Notes** |
| --- | --- | --- | --- | --- | --- | --- | --- |
| PICC | All-cause probability of CLABSI | 2% | Milstone et al., 2013 | Complication probability | Retrospective cohort study | 4797 | 149 CLABSIs in 89946 catheter days. We convert this to a probability using p = 1-exp(-149/89946*14), where 14 is the base case number of days in the model |
| PICC | Probability of a sub optimally placed catheter being too long (as opposed to too short) | 87% | Jain et al., 2013 | Complication probability | Retrospective cohort study | 319 |  |
| PICC | Probability of arrythmia | 1% | Dhillon et al., 2020 | Complication probability | Case–control study | 3180 | - Study doesn't account for patient time at risk, % reported is a simple proportion of sample - Study contains neonates and children under the age of 18 |
| PICC | Probability of cardiac tamponade | 2% | Pezzati et al., 2004 | Complication probability | Retrospective cohort study | 258 | - Study doesn't account for patient time at risk, % reported is a simple proportion of sample |
| PICC | Probability of extravasation | 4% | McIntyre et al., 2023 | Complication probability | Prospective cohort study | 25 | 1 extavasation from 377 dwell days, we convert to a probability using p = 1-exp(-rt), where r = 1/377 and t is the number of PICC dwell days (base case = 14 days) |
| PICC | Probability of migration | 28% | Acun et al., 2021 | Complication probability | Retrospective cohort study | 168 | - Study doesn't account for patient time at risk, % reported is a simple proportion of sample |
| PICC | Probability of optimal catheter position on initial insertion | 79% | Ling et al., 2019 | Complication probability | Randomised control trial | 160 | - Study doesn't account for patient time at risk, % reported is a simple proportion of sample |
| PICC | Probability of thrombosis | 9% | Park et al., 2014 | Complication probability | Review | 3332 | - Study doesn't account for patient time at risk, % reported is a simple proportion of sample |
| PICC | PICC Dwell time (days) | 14 | Milstone et al., 2013 | Resource utilisation & cost | Retrospective cohort study | 4797 | - Study reports median dwell time not mean |
| Both | Cost of treating CLABSI | 76464.78 | Goudie et al., 2014 | Resource utilisation & cost | Case-control study | 4017 |  |
| PICC | Cost of treating arrythmias | 6255.16 | Wodchis et al., 2012 | Resource utilisation & cost | Review | 27 Studies |  |
| PICC | Cost of treating cardiac tamponade | 76276.25 | Iribarne et al., 2012 | Resource utilisation & cost | Retrospective cohort study | 16788 |  |
| Both | Cost of treating extravasation | 2651.37 | Hanrahan, 2013 | Resource utilisation & cost | Simple cost comparison | n/a | - Costs estimated from assumptions |
| Both | Cost of treating thrombosis | 10548.73 | Haddad et al., 2014 | Resource utilisation & cost | Retrospective cohort study | 1030 |  |
| UVC | All-cause probability of CLABSI | 0.014 | Gibson et al., 2021 | Complication probability | Meta analysis | 14226 | 3.51 CLABSI per 1000 catheter days. We convert this to a probability using p = 1-exp(-3.51/1000*4), where 4 is the base case number of days in the model |
| UVC | Probability of UVC tip being placed too deep/far on initial insertion | 17% | Franta et al., 2017 | Complication probability | Prospective cohort study | 65 |  |
| UVC | Probability of UVC tip being positioned optimally on initial insertion | 58% | Wu et al., 2020 | Complication probability | Retrospective cohort study | 66 |  |
| UVC | Probability of extravasation | 0.1% | Gibson et al., 2021 | Complication probability | Meta analysis | 14226 | 0.13 extravasations per 1000 catheter days. We convert this to a probability using p = 1-exp(-0.13/1000*4), where 4 is the base case number of days in the model |
| UVC | Probability of migration | 14% | Gibson et al., 2021 | Complication probability | Meta analysis | 14226 | 38.09 migrations per 1000 catheter days. We convert this to a probability using p = 1-exp(-38.09/1000*4), where 4 is the base case number of days in the model |
| UVC | Probability of thrombosis | 2% | Gibson et al., 2021 | Complication probability | Meta analysis | 14226 | 5.27 migrations per 1000 catheter days. We convert this to a probability using p = 1-exp(-5.27/1000*4), where 4 is the base case number of days in the model |
| UVC | UVC Dwell time (days) | 4 | Sanderson et al., 2017 | Resource utilisation & cost |  | 1392 |  |

Table legend: PICC – Peripherally inserted central catheter; UVC – Umbilical venous catheter; CLABSI – Catheter line associated bloodstream infection
