# Supplementary material for: Umbilical venous catheter and peripherally inserted central catheter malposition and tip migration in neonates: A mixed methods cost analysis
Source: Int J Nurs Stud Adv. 2025 Nov 10;9:100450. doi: 10.1016/j.ijnsa.2025.100450 (PMC12664355; doi:10.1016/j.ijnsa.2025.100450)
Supplement: Supplementary file 3 [file mmc3.docx]

Table S2: More detailed sensitivity analyses results

| **UVC** | | | | | | | |
| --- | --- | --- | --- | --- | --- | --- | --- |
| **Variable Description** | **Variable Low** | **Variable Base** | **Variable High** | **Impact** | **Low** | **High** | **Spread** |
| Catheter dwell time | 1 | 4 | 7 | Increase | $ 245.55 | $ 578.77 | $ 333.22 |
| Probability of UVC migration | 0% | 14% | 29% | Increase | $ 343.91 | $ 439.14 | $ 95.24 |
| Daily UVC monitoring cost | $87.92 | $117.23 | $146.54 | Increase | $ 356.45 | $ 424.08 | $ 67.63 |
| Probability of thrombosis | 0% | 2% | 4% | Increase | $ 359.17 | $ 421.46 | $ 62.29 |
| Cost of accurate UVC insertion | $87.44 | $116.59 | $145.74 | Increase | $ 373.48 | $ 407.05 | $ 33.58 |
| Cost of inaccurate UVC insertion | $103.56 | $138.08 | $172.60 | Increase | $ 375.63 | $ 404.90 | $ 29.27 |
| Probability of UVC tip being positioned optimally | 52% | 58% | 63% | Decrease | $ 379.03 | $ 401.50 | $ 22.48 |
| Probability of CLABSI | 0% | 0% | 0% | Increase | $ 386.83 | $ 408.63 | $ 21.80 |
| Cost of treating CLABSI | $50,027.65 | $76,464.78 | $150,413.99 | Increase | $ 385.06 | $ 404.84 | $ 19.78 |
| Proportion of CLABSIs attributable to migration | 8% | 10% | 13% | Increase | $ 386.50 | $ 394.03 | $ 7.53 |
| **PICC** | | | | | | | |
| **Variable Description** | **Variable Low** | **Variable Base** | **Variable High** | **Impact** | **Low** | **High** | **Spread** |
| Catheter dwell time | 7 | 14 | 21 | Increase | $ 1,263.40 | $ 1,771.74 | $ 508.34 |
| Probability of migration | 21.00% | 28.00% | 36.00% | Increase | $ 1,329.04 | $ 1,733.58 | $ 404.54 |
| Daily monitoring cost | $307.73 | $410.30 | $512.88 | Increase | $ 1,409.11 | $ 1,626.55 | $ 217.44 |
| Cost of treating cardiac tamponade | $58,595.14 | $76,276.25 | $90,897.27 | Increase | $ 1,428.72 | $ 1,591.52 | $ 162.80 |
| Probability of thrombosis | 6.90% | 9.20% | 11.50% | Increase | $ 1,449.90 | $ 1,585.76 | $ 135.87 |
| Probability of optimal catheter position | 59.10% | 78.80% | 90.62% | Decrease | $ 1,475.69 | $ 1,588.07 | $ 112.38 |
| Probability of cardiac tamponade | 1.62% | 1.80% | 1.98% | Increase | $ 1,479.39 | $ 1,556.27 | $ 76.89 |
| Cost of treating CLABSI | $50,027.65 | $76,464.78 | $150,413.99 | Increase | $ 1,501.43 | $ 1,563.72 | $ 62.29 |
| Proportion of CLABSIs attributable to migration | 4.67% | 9.67% | 14.67% | Increase | $ 1,493.31 | $ 1,542.39 | $ 49.08 |
| Cost of accurate PICC insertion | $160.90 | $178.78 | $196.66 | Increase | $ 1,495.68 | $ 1,539.98 | $ 44.30 |

Table legend: PICC – Peripherally inserted central catheter; UVC – Umbilical venous catheter; CLABSI – Catheter line associated bloodstream infection; Spread = difference between model results when variable is set to its upper bound and lower bound
